# CRISPR/Cas9 in *Leishmania mexicana*: A case study of LmxBTN1

**DOI:** 10.1371/journal.pone.0192723

**Published:** 2018-02-13

**Authors:** Aygul Ishemgulova, Jana Hlaváčová, Karolina Majerová, Anzhelika Butenko, Julius Lukeš, Jan Votýpka, Petr Volf, Vyacheslav Yurchenko

**Affiliations:** 1 Life Science Research Centre, Faculty of Science, University of Ostrava, Ostrava, Czech Republic; 2 Biology Centre, Institute of Parasitology, Czech Academy of Sciences, České Budejovice (Budweis), Czech Republic; 3 Department of Parasitology, Faculty of Science, Charles University, Prague, Czech Republic; 4 University of South Bohemia, Faculty of Sciences, České Budejovice (Budweis), Czech Republic; 5 Institute of Environmental Technologies, Faculty of Science, University of Ostrava, Ostrava, Czech Republic; University of Liverpool, UNITED KINGDOM

## Abstract

*Leishmania* parasites cause human cutaneous, mucocutaneous and visceral leishmaniasis. Several studies proposed involvement of certain genes in infectivity of these parasites based on differential mRNA expression data. Due to unusual gene expression mechanism, functions of such genes must be further validated experimentally. Here, we investigated a role of one of the putative virulence factors, *LmxM*.*22*.*0010*-encoded BTN1 (a protein involved in Batten disease in humans), in *L*. *mexicana* infectivity. Due to the incredible plasticity of the *L*. *mexicana* genome, we failed to obtain a complete knock-out of *LmxM*.*22*.*0010* using conventional recombination-based approach even after ablating four alleles of this gene. To overcome this, we established a modified CRISPR-Cas9 system with genomic expression of Cas9 nuclease and gRNA. Application of this system allowed us to establish a complete BTN1 KO strain of *L*. *mexicana*. The mutant strain did not show any difference in growth kinetics and differentiation *in vitro*, as well as in the infectivity for insect vectors and mice hosts. Based on the whole-transcriptome profiling, *LmxM*.*22*.*0010*-encoded BTN1 was considered a putative factor of virulence in *Leishmania*. Our study suggests that ablation of *LmxM*.*22*.*0010* does not influence *L*. *mexicana* infectivity and further illustrates importance of experimental validation of *in silico-*predicted virulence factors. Here we also describe the whole genome sequencing of the widely used model isolate *L*. *mexicana* M379 and report a modified CRISPR/Cas9 system suitable for complete KO of multi-copy genes in organisms with flexible genomes.

## Introduction

Of the 53 *Leishmania* species described to date, more than twenty can infect humans and domestic animals [1] and cause leishmaniases–several associated diseases affecting populations of almost one hundred countries mostly in tropical and subtropical regions [2,3]. The parasites are primarily transmitted by blood sucking females of phlebotomine sand flies [4,5] and clinical pleomorphism range from innocuous often self-healing skin lesions to potentially fatal visceral organ failures. Early diagnosis, access to treatment, vector control and the containment of epidemic outbreaks are still challenging problems in endemic areas, especially for visceral leishmaniasis, one of the most neglected poverty-related diseases [1]. To better understand the complicated epidemiology of this disease and develop new treatment and preventative strategies, one needs to investigate factors which influence *Leishmania* growth and differentiation in both sand fly vectors and vertebrate hosts.

The life cycle of *Leishmania* comprises two main developmental forms–extracellular promastigotes within the alimentary tract of the vector, and intracellular amastigotes residing in the phagolysosomal vacuoles of their vertebrate host's phagocytic cells [6,7]. Genes and proteins involved in differentiation or infection maintenance are putative virulence factors. Nowadays, they are identified mainly by using NGS-based approaches [8,9] and analyzed with the help of functional genomics methods [10–12].

One of the genes identified in our previous bioinformatics analyses based on transcriptional profiles and phyletic patterns, was *LmxM*.*22*.*0010*. Its orthologues were shown to be upregulated in *L*. *mexicana* M379 amastigotes as compared to corresponding promastigotes, and a virulent strain of *L*. *major* LV561 had higher level of its expression as compared to its avirulent counterpart [9]. We considered the product of this gene as a protein of unknown function potentially involved in *Leishmania* virulence and investigated it here further by *in silico*, *in vitro*, and *in vivo* approaches.

In *L*. *mexicana*, *LmxM*.*22*.*0010* encodes a homolog of BTN1, a protein involved in Batten disease in humans, also known as YHC3P and CLN3 in other organisms. This protein is needed for the ATP-dependent transport of arginine into the vacuole, a process that also requires a functional vacuolar H+-ATPase (V-ATPase) complex [13]. Since the yeast deletion strain for *btn1* displays an abnormally acidic vacuolar pH during early growth phases, it is considered to play a role in vacuolar pH maintenance [14,15] and distribution of phospholipids in cell membranes [16]. The human homolog of BTN1 is a lysosomal transmembrane protein, mutations in which cause Batten disease, the juvenile form of neuronal ceroid lipofuscinosis characterized by a decline in mental abilities, loss of motor skills, blindness, epileptic seizures, and premature death [17,18].

In this work we established an efficient CRISPR-Cas9 system and demonstrated that despite being upregulated in virulent isolates or infectious developmental stages of *Leishmania*, *LmxM*.*22*.*0010* is not essential for virulence or development of these parasites *in vitro* and *in vivo*.

## Materials and methods

### *In silico* analyses

For the identification of the *LmxM*.*22*.*0010* homologs, a BLASTP search was performed using this protein as a query and TriTryp v.9.0 as a database [19]. Amino acid sequences of all identified proteins with the sequence percent identity to the query higher than 35% were aligned using Muscle v. 3.8.31 with default parameters [20]. Poorly aligned regions of the alignment were removed using Gblocks [21] with the following parameters: -b3 = 10; -b4 = 2; -b5 = h. Maximum likelihood phylogenetic tree was inferred using the IQTREE multicore v.1.5.3 with JTT + G4 model [22]. Branch supports were assessed by bootstrapping with 1,000 standard replicates. Bayesian inference of phylogeny was conducted using MrBayes 3.2.6 with analysis run for 1 million generations, sampling every 100^th^ of them [23]. The model of site heterogeneity was set based on IQTREE analyses (G4), while the optimal model of amino acid substitutions (Jones) was found by MrBayes using mixed amino acid model prior.

### *Leishmania* parasites axenic cultivation, growth, and differentiation

*Leishmania mexicana* (isolate MNYC/BZ/62/M379) culture was maintained in M199 medium (Sigma-Aldrich, St. Louis, USA) supplemented with 2 μg/ml Biopterin (Sigma-Aldrich), 2 μg/ml Hemin (Jena Bioscience GmbH, Jena, Germany), 25mM HEPES, 50 units/ml of penicillin, 50 μg/ml of streptomycin and 10% Fetal Bovine Serum, FBS (all from Life Technologies, Carlsbad, USA) at 23°C. Both BTN1 knock-out (KO) and WT *L*. *mexicana* were passaged through insect vectors and mice prior to *in vitro* analyses. Growth kinetics comparison was performed for 6 days from the starting density of 5 × 10^5^ parasites per ml. Cell numbers were counted using a hemocytometer every 48 hours as described previously [24] in four biological replicates.

*In vitro* differentiation was performed as described elsewhere by varying pH and temperature [25] with modifications [10]. The genes encoding PFR1D (*LmxM*.*08_29*.*1750*, *LmxM*.*08_29*.*1760*), SHERP (*LmxM*.*23*.*1050*, *LmxM*.*23*.*1061*) and Amastin (*LmxM*.*08*.*0800*, *LmxM*.*08*.*0840*, *LmxM*.*08*.*0850*) were used as promastigotes- (procyclics and metacyclics), metacyclics- and amastigotes-specific markers, respectively [26,27]. For normalization, expression values of *LmxM*.*07*.*0510* (gene encoding a 60S ribosomal protein L7a) and *LmxM*.*36*.*1140* (gene encoding a short chain 3-hydroxyacyl-CoA dehydrogenase) were used [28]. Quantitative PCR analysis (RT-qPCR) was performed as described previously [29]. Primer sequences for RT-qPCR are listed in the S1 Table.

### Genome sequencing and analysis

The genome of *L*. *mexicana* isolate MNYC/BZ/62/M379 was sequenced as describe previously [24,30] using the Illumina HiSeq technology at Macrogen Inc. (Seoul, South Korea). It is uploaded to the TriTryp database [19] under accession number SRP126412. The average gene coverage was calculated in CLC Genomics Workbench v. 7.0 (Qiagen, Hilden, Germany) after the mapping of the genomic reads with the following parameters: mismatch cost, 2; insertion cost, 2; deletion cost, 2; length fraction, 0.8; similarity fraction, 0.8; and the rest set to default.

### Genetic manipulations in *Leishmania mexicana*

To ablate *LmxM*.*22*.*0010* in *L*. *mexicana*, all alleles were sequentially replaced with selectable markers for Nourseothricin (Sat), Hygromycin (Hyg), Neomycin (Neo) and Bleomycin (Ble). Targeting constructs were generated by fusion PCR [31]. In the first round of PCR, 5' and 3' arms of homology were amplified from the *L*. *mexicana* genomic DNA using primers A/B (C, I, K) and D (E, J, L)/F, respectively (S1 Table). The ORFs of the Sat, Hyg, Neo and *Ble*-resistance genes were amplified from the plasmids pF4T7polNLS1.4sat and pF4TR1.4hyg, pTUB_APegfp_dN_1.4kIRneo [32], pLEXSY-ble2 (Jena Bioscience) using primers SAT_5'f/ SAT_3'r and Hyg_5'f/ Hyg_3'r, NEO_5'f/ NEO_3'r and BLE_5'f/ BLE_3'r (S1 Table). In the fusion PCR reaction, 5' and 3' arms of homology were combined with either Sat, Hyg, Neo or Ble resistance gene and amplified with nested primers G and H (S1 Table). *L*. *mexicana* promastigotes were transfected with 5 μg of the targeting constructs as described previously using BTX ECM 630 electroporator (Harvard Apparatus Inc, Holliston, USA) [33]. The first allele knockout cell line was isolated in complete M199 medium containing 100 μg/ml of Sat (Jena Bioscience). The second allele knockout *L*. *mexicana* clones were selected on solid M199 medium supplemented as above with additional 100 μg/ml of Sat and 100 μg/ml of Hyg. The third allele knockout *L*. *mexicana* clones were selected on solid M199 medium supplemented as above with additional 100 μg/ml of Sat, 100 μg/ml of Hyg, and 50 μg/ml of Neo. The forth allele knockout *L*. *mexicana* clones were selected on solid M199 medium supplemented as above with additional 100 μg/ml of Sat, 100 μg/ml of Hyg, 50 μg/ml of Neo and 100 μg/ml of Ble. Correct integration was confirmed by PCR on genomic DNA with specific primers and by Southern blot [34]. In brief, total genomic DNA was isolated using DNeasy Blood & Tissue Kit (Qiagen, Hilden, Germany), digested with *Bst*EII overnight, separated on 0.75% agarose gel, and transferred to a ZetaProbe blotting membrane (Bio-Rad, Hercules, USA). Blots were blocked and hybridized with ^32^P-labeled PCR probes for Sat, Hyg, Neo, Ble, 5' UTR and ORF of *LmxM*.*22*.*0010* gene. The following primers (S1 Table) were used to amplify probes: SBp_SAT_f and SBp_SAT_r (for Sat), SBp_Hyg_f and SBp_Hyg_r (for Hyg), SBp_Neo_f and SBp_Neo_r (for Neo), SBp_Ble_f and SBp_Ble_r (for Ble), SBp_*LmxM*.*22*.*0010*_5'f and SBp_*LmxM*.*22*.*0010*_5'r (for 5' UTR), SBp_*LmxM*.*22*.*0010*_f and SBp_*LmxM*.*22*.*0010*_r (for *LmxM*.*22*.*0010* ORF). Probes were labeled with radioactive ^32^P using the DecaLabel DNA Labeling kit (Thermo Fisher Scientific, Waltham, USA).

### CRISPR-Cas9 in *L*. *mexicana* verification: mCherry

Humanized *Streptococcus pyogenes* Cas9 nuclease with nuclear localization sequence was amplified from PX330a plasmid [35] and cloned into pLEXSY-hyg2 (Jena Bioscience) plasmid. As a proof of principle, the functionality of the system was first tested on cells expressing a fluorescent protein. *L*. *mexicana* expressing mCherry [28] was transfected with 5 μg of linearized resulting plasmid and clones of *L*. *mexicana*-mCherry-Cas9 were selected on solid M199 medium supplemented as above with additional 100 μg/ml of Hyg. *L*. *mexicana* U6 promoter and terminator sequences were determined based on previous work and amplified from the genomic DNA [36]. gRNA for mCherry open reading frame (taactttccatctggcggcc|cgg) was selected using eukaryotic pathogen CRISPR guide RNA/DNA Design Tool at http://grna.ctegd.uga.edu [37]. U6 promoter in forward orientation, gRNA with tracrRNA and U6 terminator were amplified with the primers: A_U6_prom_LmxM_f and B_U6_prom_LmxM_r, E_sgRNA_mCh396_f and F_sgRNA_mCh396_r, C_U6_term_LmxM_f and D_U6_term_LmxM_r, respectively. Later they were merged together by fusion PCR with primers G_sgRNA_mCh396_f and H_sgRNA_mCh396_r (S1 Table). Donor sequence encoding Puromycin (Puro) resistance gene was amplified from the pLS6-PFR2 [38] with 30 bp of mCherry sequences flanking double stranded break site using primers I_donor_mCh_f and J_donor_mCh_r. *L*. *mexicana*-mCherry-Cas9 cells were transfected with 5 μg of gRNA and 5 μg of donor amplicons. Parasites were selected on liquid M199 medium supplemented as above with additional 100 μg/ml of Hyg, 100 μg/ml of Sat, and 20 μg/ml of Puro. Ablation of mCherry was checked by fluorescent microscopy and PCR with primers mCherry_F_NcoI and mCherry_R_NotI (mCherry size is 744 bp; after donor insertion size is 2,354 bp).

### CRISPR-Cas9 in *L*. *mexicana*: Ablation of *LmxM*.*22*.*0010*

Wild type of *L*. *mexicana* was tansfected with 5 μg of the linearized plasmid Cas9/pLEXSY-hyg2 and individual clones Cas9 were selected as above on solid M199 medium supplemented with 100 μg/ml of Hyg. In order to ablate *LmxM*.*22*.*0010* we used the same principal strategy as for the mCherry knock-out with gRNA gatgttcgcccacgtgataa|ggg. The U6 promoter in forward orientation, gRNA with tracrRNA and U6 terminator were amplified using the following primers: A_U6_prom_LmxM_f and B_*LmxM*.*22*.*0010*-sgRNA348_r, C_U6_term_ *LmxM*.*22*.*0010*_f and D_U6_term_ *LmxM*.*22*.*0010*_r, E_ *LmxM*.*22*.*0010*-sgRNA348_f and F_sgRNA_mCh396_r, respectively. These fragments were fused with primers G_sgRNA_NotI_f and H_sgRNA_NcoI_r and cloned into pLEXSY-SAT2 (Jena Bioscience). Donor sequence encoding Puro resistance was amplified from pLS6-PFR2 [38] with 30 bp of *LmxM*.*22*.*0010* sequences flanking double stranded break site using primers I_donor_*LmxM*.*22*.*0010*–348_f and J_donor_*LmxM*.*22*.*0010*–348_r (S1 Table). Construct containing gRNA and donor construct were transfected into the wild type *L*. *mexicana*. Clones were selected on solid M199 medium supplemented as above with additional 100 μg/ml of Hyg, 100 μg/ml of Sat, and 20 μg/ml of Puro. Ablation of *LmxM*.*22*.*0010* was verified by PCR with primers qPCR_*LmxM*.*22*.*0010*_f and qPCR_*LmxM*.*22*.*0010*_1r (wild type *LmxM*.*22*.*0010* size is 515 bp; after donor insertion the size is 1,765 bp), qPCR with primers qPCR_*LmxM*.*22*.*0010*_f and qPCR_*LmxM*.*22*.*0010*_ar and by Southern blot as above with probes 5' UTR-, 3' UTR-, and Puro. The following primers were used for Puro probe amplification: South_Puro_f and South_Puro_r, other primers were as above.

### Infection of sand fly vectors

Development of wild type of *L*. *mexicana* (WT), Cas9 clones (Cas9), and strain with ablated *LmxM*.*22*.*0010* (BTN1 KO) was studied in laboratory colony of *Lutzomyia longipalpis* (strain Jacobina originated from Bahia State, Brazil). Sand flies were maintained under standard conditions as described previously [39]. Suspensions of heat-inactivated rabbit blood and promastigotes (10^6^ cells per ml) were prepared in sterile conditions and sand fly females were fed through a chicken skin membrane. Blood-fed females were separated and dissected on days 2–3 and 7–8 post infections (p.i.) to analyze localization and intensity of infection under a light microscope. Intensity of infection was considered as light (< 100 parasites/gut), medium (100–1,000 parasites/gut), and heavy (> 1,000 parasites/gut) as described previously [40]. The experiment was repeated five times.

In three out of five experiments, smears of dissected guts (day 7–8 p.i.) were prepared from three randomly chosen blood-fed females for every strain (27 females in total). Smears were air dried, fixed by methanol, stained by Giemsa (Sigma-Aldrich), and used for morphological analysis of *Leishmania* parasite cells under the Olympus BX51 light microscope equipped with a DP72 CCD camera (Olympus, Tokyo, Japan). Within each smear, 60 randomly selected cells (1,620 in total) were measured for three parameters–length and width of the cell body, and length of the flagella–using QuickPHOTO micro v. 3.0 (Promicra, Prague, Czech Republic). Three predominant *Leishmania* morphotypes were distinguished and categorized as described previously [41] with the following modifications: long nectomonads body length ≥ 12 μm; short nectomonads body length < 12 μm; and metacyclic promastigotes body length ≤ 8 μm and flagella/body ratio > 1.5.

### Mice infection

To mimic natural infections, BALB/c mice were inoculated by midgut content of *L*. *longipalpis* females infected by *Leishmania* [42]. On days 7–8 p.i., sand fly females were checked by dissection of guts under a stereomicroscope to confirm heavy infection with colonization of the stomodeal valve (= mature infection). Dissected thoracic midguts with high parasite density were pooled separately for different *Leishmania* strains, homogenized in sterile saline solution and immediately injected intra-dermally into the ear pinnae of ketamine/xylazin anesthetized BALB/c mice (females, ~ 3 months old). The injected 5 μl volume of the homogenate corresponds to five thoracic midguts per mice. Development of clinical symptoms (size of nodular lesions) was monitored weekly.

In the first experiment, five mice for each *Leishmania* strain (WT, Cas9, and BTN1 KO) were used and sacrificed at the 15^th^ week p.i. In the second experiment, mice infected with WT (n = 3), Cas9 (n = 4), and BTN1 KO (n = 4) *L*. *mexicana* were sacrificed at the 16^th^ week p.i. Parasites isolated from these mice were passaged through sand flies and used to infect mice in the third experiment: WT (n = 3), Cas9 (n = 4), and BTN1 KO (n = 5). Because the clinical manifestations were worse than in the previous two experiments and to avoid unnecessarily suffering, in the third experiment animals were sacrificed at the 13^th^ week p.i. Mice were dissected and their infected ears were used for both *Leishmania* re-isolation by cultivation and quantification of parasite load by qPCR.

#### Ethics statement

Animals were maintained and handled in the animal facility of Charles University in Prague in accordance with institutional guidelines and Czech legislation (Act No. 246/1992 and 359/2012 coll. on protection of animals against cruelty in present statutes at large), which complies with all relevant European Union and international guidelines for experimental animals. All the experiments were approved by the Committee on the Ethics of Laboratory Experiments of the Charles University in Prague and were performed under permission No. MSMT-31114/2015-13 of the Ministry of the Environment of the Czech Republic. Investigators are certificated for experimentation with animals by the Ministry of Agriculture of the Czech Republic. All efforts were made to minimize the number and the suffering of experimental animals during the study.

### Quantitative PCR analysis of parasites from insects and mice

Extraction of total DNA from homogenized mice ear tissues and sand flies was performed using a DNA isolation kit for cells and tissue (Roche Diagnostics, Indianapolis, USA) according to the manufacturer’s instructions. To quantify the numbers of *Leishmania* parasites in the guts of sand fly females (7–8 days p.i.) and in the inoculated ear of infected mice (13–16 weeks p.i.), the qPCR with *Leishmania* kinetoplast DNA-specific primers was performed using the iQ SYBR Green Supermix in Bio-Rad iCycler & iQ Real-Time PCR Systems (Bio-Rad) as described previously [40]. Log-transformed data were evaluated using Statistica v. 6.1 (TIBCO Software Inc., Palo Alto, USA).

## Results and discussion

### *LmxM*.*22*.*0010 in silico* analyses

The returned BLAST hits for *LmxM*.*22*.*0010* clearly denoted its homology to BTN1/CLN3-encoding genes in other organisms: *Strigomonas culicis cln3*/*btn1* (E-value 0), *Oxytricha trifallax cln3*/*btn1* (E-value 7e^-32^), *Homo sapiens* battenin (E-value 3e^-7^). As we previously reported, this gene was present at the basal node of Trypanosomatidae and then lost is some lineages, including *Paratrypanosoma confusum*, *Trypanosoma cruzi*, *T*. *vivax*, *T*. *congolense* and others [9]. This prompted us to investigate its phyletic distribution in Leishmaniinae [43] in more detail. Indeed, applying the 35% identity threshold, this gene was readily detectable in virtually all analyzed species with sequenced genomes (S2 Table and S1 Fig). The noticeable exception is a lizard parasite, *Leishmania tarentolae* [44]. This can be explained by the fact that the contigs of the syntenic region containing a putative orthologue of this gene, were assembled poorly in this species. Of note, one of the "gold standards" in *Leishmania* research, a model isolate Friedlin of *Leishmania major*, has two identical paralogs of *LmxM*.*22*.*0010* located on chromosome 6 (*LmjF*.*06*.*1300*) and chromosome 22 (*LmjF*.*22*.*0010*) (S2 Table and S1 Fig).

We sequenced the genome of *L*. *mexicana* M379 and detected 40,850 single nucleotide variants differed it from the reference *L*. *mexicana* genome. These data were used to check for the potential off-target binding sites for designed gRNA molecules. Our ploidy analysis of the chromosome 22 revealed that *LmxM*.*22*.*0010* is duplicated in this species. Its average gene coverage was approximately twice as high as average coverage of other genes on the same chromosome (S3 Table). We concluded that *LmxM*.*22*.*0010* in the isolate M379 is present in two copies.

### Conventional genetic ablation of *LmxM*.*22*.*0010*

**Fig 1:**
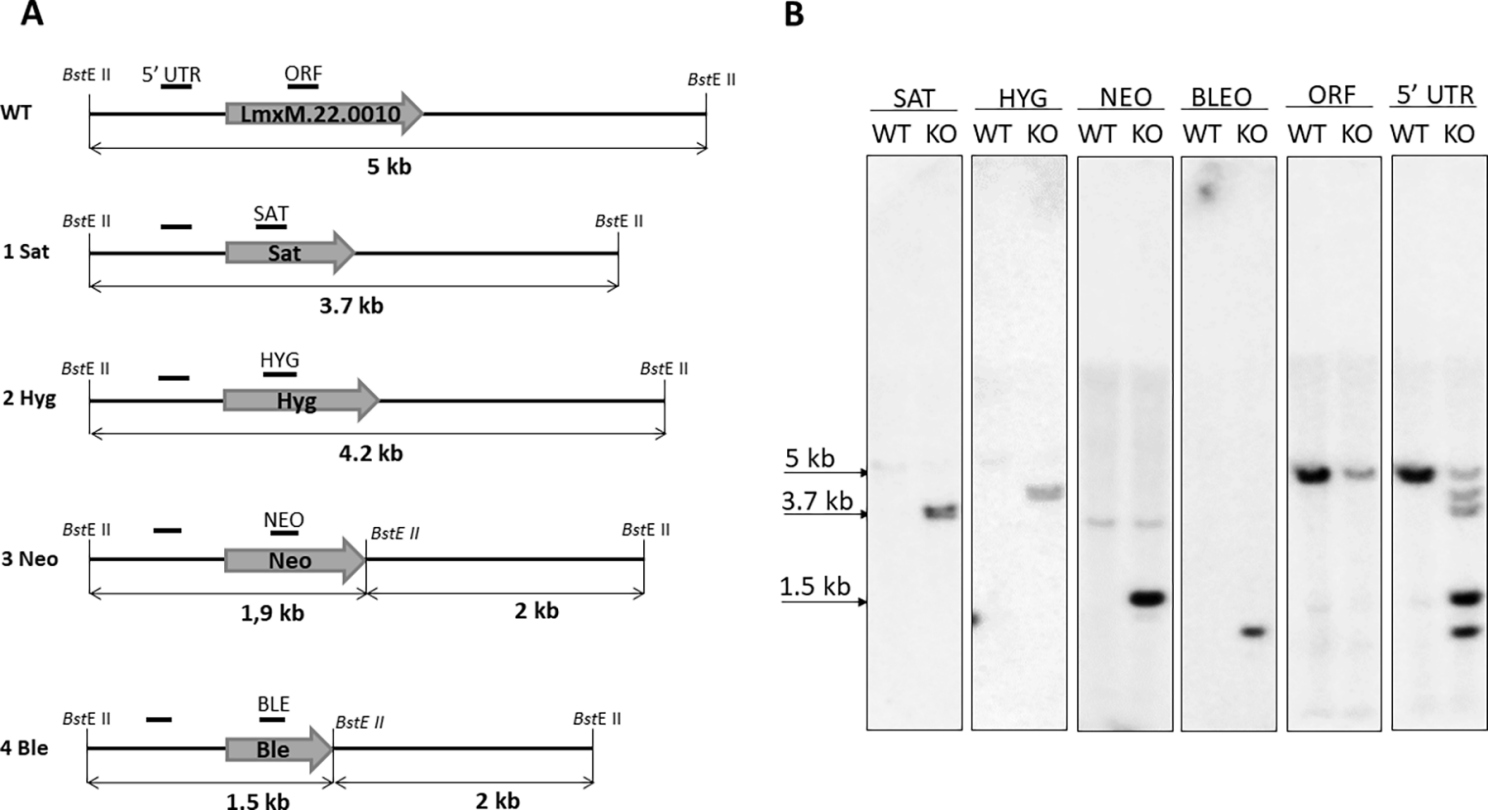
Ablation of *LmxM*.*22*.*0010* by conventional approach. A, Schematic representation of the WT and recombined alleles after replacement with Sat-, Hyg-, Neo-, and Ble-resistant genes. Annealing positions of the probes and expected fragment sizes are shown. B, Southern blot analysis of the *Bst*E II digested *L*. *mexicana* genomic DNA of the WT and BTN1 ablated strains with Sat, Hyg, Neo, Ble, 5' UTR, and *LmxM*.*22*.*0010* ORF probes.

To investigate involvement of *LmxM*.*22*.*0010* in *L*. *mexicana* virulence, we subsequently ablated all 4 alleles of this gene by replacing them with antibiotic resistance genes for Sat, Hyg, Neo, and Ble (Fig 1). To our surprise, Southern blotting results showed that even after four copies of this genes were successfully replaced, *LmxM*.*22*.*0010* knock-out was not complete (Fig 1B, panel 22.0010). We concluded that there must be at least one additional copy present elsewhere in the *L*. *mexicana* genome. Whether this is a result of genetic manipulations or occurs naturally remains to be investigated further. This phenomenon illustrates an incredible plasticity of the *L*. *mexicana* genome [45] and highlights urgent need for developing novel approaches to *Leishmania* genetics.

### Establishing and validation of the efficient CRISPR-Cas9 system in *L*. *mexicana*

The CRISPR-Cas9 genome editing methodology has been successfully applied in *Leishmania* and *Trypanosoma* spp. All the established systems rely on different methods of gRNA delivery: transfection of *in vitro* transcribed gRNA [46,47], expression of gRNA from the plasmid under the control of the RNA polymerase I promoter and hepatitis delta virus ribozyme [48,49], or U6 promoter and terminator [38]. In trypanosomatids, double stranded DNA breaks produced by Cas9 are usually repaired by microhomology-mediated end joining. If donor sequence is provided, such breaks are repaired by homologous recombination [46]. In order to implement selection, a donor construct with antibiotic resistance is usually co-transfected along with *Cas9* and gRNA molecules. Online tools for gRNA [37] and primer design [47] are readily available.

Because of its high efficiency and relative simplicity, CRISPR-Cas9 high-throughput methods for gene tagging and ablation in *Leishmania* were recently proposed. Nevertheless, all the systems described thus far have certain limitations. The main one is an episomal (plasmid) or PCR-product source of the *Cas9* gene. It may easily be lost during prolonged cultivation of *L*. *mexicana* [47,50]. To overcome this, we established an efficient CRISPR-Cas9 system in *L*. *mexicana* that combines features of several previously reported systems [37,38,46]. The main distinctions are: (i) ribosomal DNA locus integrated Cas9-coding sequence; (ii) gRNA expression under the control of native *L*. *mexicana* U6 promoter and terminator. Proper integration of all constructs was confirmed by PCR.

We first validated the system ablating *mCherry* from the *L*. *mexicana-mCherry* strain established previously [28]. We used native *L*. *mexicana* U6 promoter and terminator to control gRNA expression. Because of the bi-directional nature of the U6 promoter [51], we tested it in both forward and reverse orientations. gRNA was designed using the Eukaryotic Pathogen CRISPR guide RNA/DNA design tool [37]. Donor sequence for integration and disruption of the target gene's ORF contained Puro resistance gene (S2A Fig). After simultaneous transfection of gRNA and donor constructs, *Leishmania* populations were selected in liquid medium supplemented with Sat, Hyg and Puro. PCR and fluorescent microscopy analyses showed complete ablation of *mCherry* ORF with U6 promoter in either forward or reverse orientation (S2 Fig). The efficacy of the system was extremely high, as we did not observe any red cell after selection under the microscope (S2D Fig) and did not detect a band corresponding to the unmodified *mCherry* by PCR (S2B Fig). In summary, we have established an efficient CRISPR-Cas9 system for gene editing in *L*. *mexicana*.

### Genetic ablation of *LmxM*.*22*.*0010* using CRISPR-Cas9

**Fig 2:**
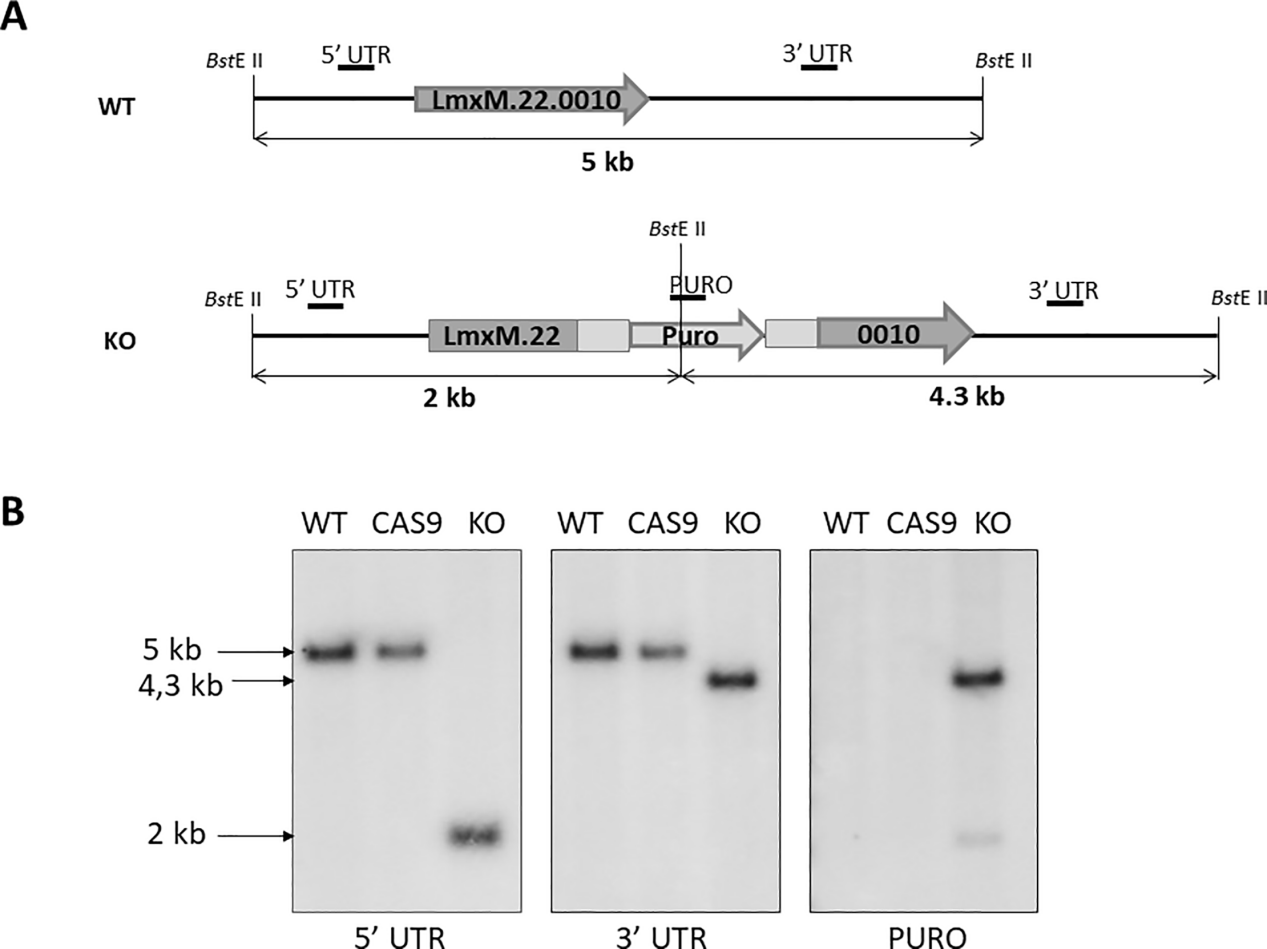
Ablation of *LmxM*.*22*.*0010* by CRISPR-Cas9. A, Schematic representation of the WT and recombined alleles after replacement with Puro resistant gene. Annealing positions of the probes and expected fragment sizes are shown. B, Southern blot analysis of the *Bst*E II digested *L*. *mexicana* genomic DNA of the WT, Cas9, and BTN1 ablated strains (labeled KO) with *LmxM*.*22*.*0010* 5' UTR, *LmxM*.*22*.*0010* 3' UTR and Puro probes.

In order to overcome difficulties with *LmxM*.*22*.*0010* conventional knock-out, we decided to use our CRISPR-Cas9 system to ablate this gene in *L*. *mexicana*. The gRNA under control of the U6 promoter in forward orientation was stably expressed from the *L*. *mexicana* 18S rDNA locus. Southern blotting (Fig 2), PCR and qRT-PCR data (S3 Fig) demonstrated complete ablation of the *LmxM*.*22*.*0010* gene. Similarly to the case of *mCherry*, the efficacy of our CRISPR-Cas9 system was extremely high, as we did not detect a band corresponding to the wild type *LmxM*.*22*.*0010* by PCR (S3B Fig). The resulting strain was named BTN1 KO. As a control for all subsequent experiments, we have also created a line expressing the Cas9 nuclease only and checked it in the same way (Fig 2).

### BTN1 KO *L*. *mexicana* grows and develops normally *in vitro*

We first investigated the effect of Cas9 expression and *LmxM*.*22*.*0010* ablation on *L*. *mexicana* growth by comparing cell division kinetics of WT, Cas9 and BTN1 KO strains *in vitro*. To exclude the negative effect of continuous cultivation [52], procyclic promastigote cultures were started from *Leishmania* cells that had previously been passaged first through insects and then mice. Cell growth monitored every 48 hours in a continuously growing culture revealed that Cas9 and BTN1 KO strains grew at the same rate as WT till the day 8 of the culture (S4 Fig). In the late stages of culturing (day 10), both Cas9 and KO strains exhibited slower growth rates compared to the WT (doubling times approximately 44, 47, and 32 hours, respectively). We assume that Cas9 expression may slightly inhibit *L*. *mexicana* growth similarly to the situation documented in *Trypanosoma cruzi* [46]. This contrasts one of the recent papers on CRISPR-Cas9-mediated gene ablation in *L*. *mexicana* [47]. The differences in the system design (integrated versus transient expression of Cas9) and time points analyzed (10 days versus 4 days) may account for such a discrepancy.

The ability to complete a life cycle is of ultimate importance for *Leishmania*. The transition from the procyclic through metacyclic promastigote to the amastigote stage can be reproduced in an axenic culture [25]. To investigate whether expression of Cas9 and ablation of *LmxM*.*22*.*0010* affected *L*. *mexicana* development, WT, Cas9 and BTN1 KO cells were differentiated *in vitro*. qRT-PCR expression analysis of previously identified stage-specific markers (*Pfr1d* for procyclic and metacyclic promastigotes, *Sherp* for metacyclic promastigotes, and *Amastin* for amastigotes [26,27]) confirmed normal development of Cas9 and BTN1 KO strains *in vitro* (S5 Fig). We noted an elevated *Amastin* expression in BTN1 KO amastigotes and explain it by compensatory mechanisms these cells must engage upon differentiation. Comparison of the measured variables (cell size and length of flagella; ANOVA; Statistica v. 6.1) showed no significant differences between the tested strains of parasites (WT, Cas9 and BTN1 KO).

We conclude that ablation of *LmxM*.*22*.*0010* does affect neither cell division rate, nor differentiation and morphology of *L*. *mexicana in vitro*.

### Ablation of *LmxM*.*22*.*0010* has no effect on *L*. *mexicana* development in sand flies and mice

**Fig 3:**
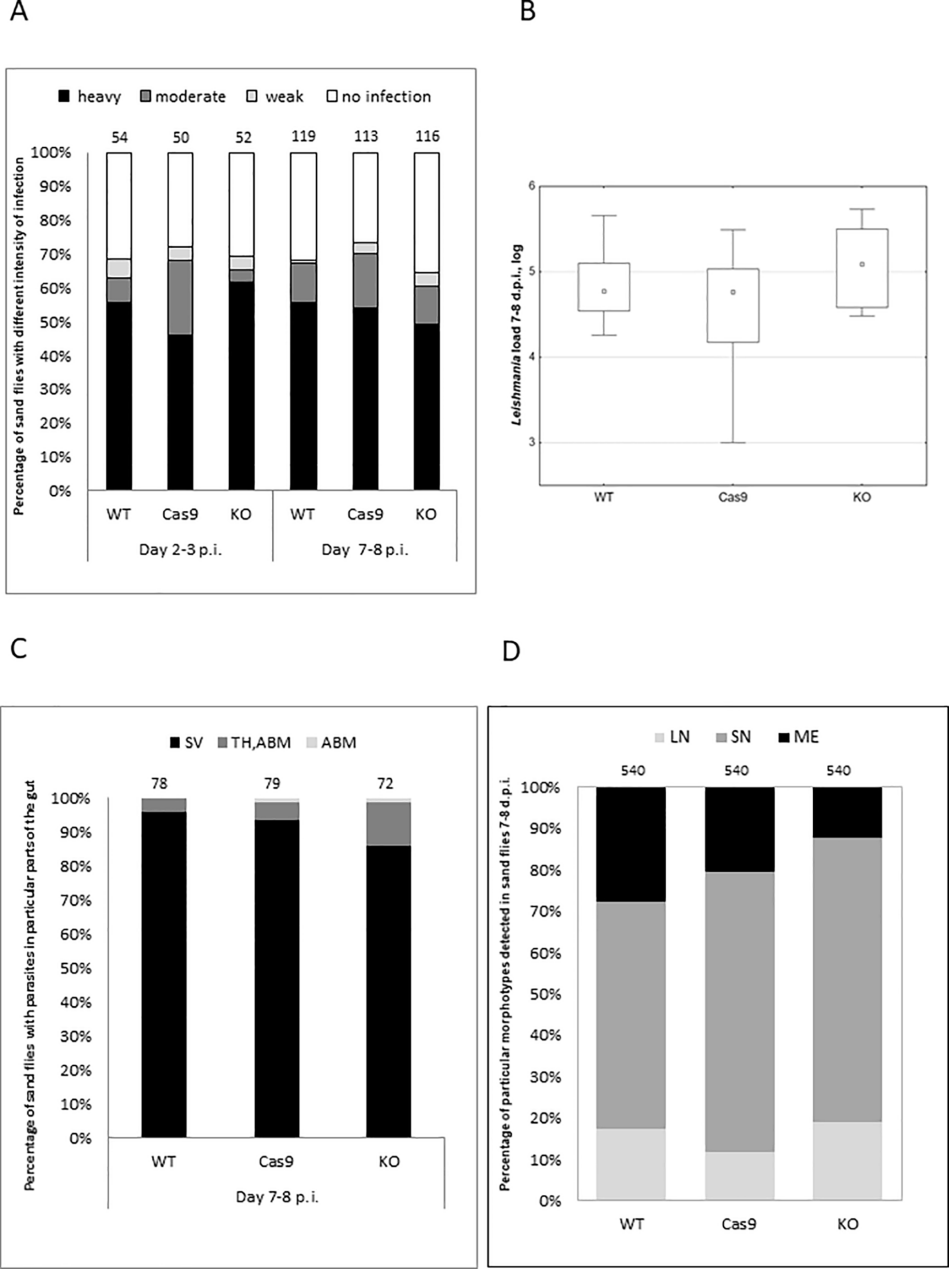
Development of the WT, Cas9 and BTN1 KO strains (labeled KO) in sand flies. A, Intensity of infection was assayed on days 2–3 and 7–8 p.i. and defined as weak (less than 100 promastigotes), moderate (100–1,000 promastigotes), or heavy (over 1,000 promastigotes), depending on the number of parasites per gut. Data are summarized from five independent biological replicates, numbers above each bar indicate the total number of dissected females. B, Quantitative PCR analysis of the *L*. *mexicana* load in the insect gut 7–8 days p.i. Boxplots are from five independent biological replicates and show 1st quartile, median, 3rd quartile, and 1.5× interquartile range values. C, Localization of parasites in sand fly gut 7–8 days p.i. (SV, stomodeal valve; TH,ABM, both thoracic and abdominal midgut; ABM, abdominal midgut). Numbers above each bar indicate the number of dissected females. D, Morphological analysis of *Leishmania mexicana* cells from thoracic midgut and stomodeal valve of infected sand fly females 7–8 days p.i. (LN, long nectomonade; SN, short nectomonade; ME, metacyclic promastigote).

Infection rate, parasite load and localization of three strains (WT, Cas9 and BTN1 KO) were compared 2–3 and 7–8 days p.i. in *Lutzomyia longipalpis* females in five independent experiments. Although we noticed slight differences in infection rate within as well as between repetitions, the ability to establish mature infection was confirmed for all three strains in each of the five independent experiments. Statistical analysis based on the pooled data from all five independent experiments showed no significant differences in the intensity of infection and localization of parasites in sand fly guts between the tested strains (Fig 3A). These data were confirmed by qPCR (Fig 3B). The comprehensive statistical analysis of the quantitative PCR did not revealed any significant differences (F(2;143) = 0.77; p = 0.46) in numbers of parasites 7–8 days p.i. in sand flies infected by WT, Cas9 and BTN1 KO *L*. *mexicana* strains.

No significant differences were observed in the parasite localization in sand fly guts, despite the fact BTN1 KO colonized less frequently the stomodeal valve (~ 10% difference) compared to other two strains (Fig 3C). Morphological analysis of *Leishmania* cells obtained from infected sand flies dissected 7–8 days p.i. has showed lower proportion of metacyclic promastigotes (ME) in BTN1 KO strain compared to the WT and Cas9 counterparts (Pearson's χ^2^ test: 31.04; df = 2, p < 0.0001) (Fig 3D). Metacyclic promastigotes are important life stages of *Leishmania*, as they are virulent for the vertebrate host. On the other hand, the developmental precursors of metacyclic promastigotes, short nectomonads (SN in Fig 3D), were more frequent in sand flies infected by KO and Cas9 compared to those infected by WT (Pearson's χ^2^ test: 12.08; df = 2, p < 0.001) (Fig 3D and S4 Table). The third form, long nectomonads (LN in Fig 3D), constituted similar percentage in all three strains. Differences in proportion of short nectomonads and metacyclics can be explained by attenuated growth observed in KO and BTN1 KO *L*. *mexicana* lines at later time points (S4 Fig). Nevertheless, our data indicate that ablation of *LmxM*.*22*.*0010* does not affect the ability of *L*. *mexicana* to establish mature infection in *Lutzomyia longipalpis* and has no influence on parasite abundance in the insect vector. Slightly delayed formation of metacyclic promastigotes and colonization of the stomodeal valve, found in BTN1 KO strain, is probably caused by its slower growth rate.

To investigate parasite development in BALB/c mice, three independent experiments were performed. In the first and second experiments, the parasites of all three tested strains used for mice infections were passaged in culture prior the infection. In the third experiment, *Leishmania* parasites used for infection originated from mice and were not cultivated *in vitro* for more than 1 day.

All mice inoculated by WT, Cas9 and BTN1 KO strains developed lesions. In WT and Cas9 strains, size of lesions did not significantly differ within, as well as between, three independent experiments. In BTN1 KO strain, first two experiments demonstrated slower development of the ear lesions (S6 Fig), and significantly smaller diameter of the lesions compared to the WT and Cas9 (F(1;24) = 19.37; p = 0.002). However, in the third experiment in mice, the lesions caused by BTN1 KO strain did not significantly differ in diameter from those produced by WT and Cas9 (F(1;10) = 1.24; p = 0.30, S6 Fig). We explain this discrepancy by constraints imposed on *Leishmania* by *in vitro* cultivation. Even several passages in culture may make the parasites less fit compared to their freshly isolated kins. Importantly, we confirmed that Cas9 gene was not lost upon prolonged cultivation of Cas9 and BTN1 KO *Leishmania* in mice.

Quantitative PCR analysis of the inoculated ears (infected mice sacrificed 13 to 16 weeks p.i.) showed high numbers of *Leishmania* parasites in all three groups (WT, Cas9 and BTN1 KO). Although the number of parasites varies considerably between individual mice, both within and between the three independent experiments, differences between WT, Cas 9 and BTN1 KO strains were not significant (F(2;35) = 1.54; p = 0.23) (S7 Fig). The number of parasites positively correlated (p = 0.05) with the lesion size measured at the end of the experiments (S8 Fig).

## Conclusions

The homolog of BTN1, a protein involved in Batten disease in humans, is responsible for ATP-dependent transport of arginine into the vacuole and implicated in vacuolar pH maintenance and distribution of phospholipids in cell membranes. It is overexpressed in the virulent isolate of *L*. *major* (compared to the avirulent one) and in the infectious amastigotes of *L*. *mexicana* (compared to the promastigotes). Based on its expression pattern we hypothesized that this protein might be involved in governing *Leishmania* virulence. However, the *L*. *mexicana* BTN1 KO strain is as infectious *in vivo* as its wild-type counterpart.

In the process of ablating the BTN1-encoding *LmxM*.*22*.*0010* we sequenced the genome of the routinely used isolate of *L*. *mexicana* (MNYC/BZ/62/M379) and established an efficient CRISP-Cas9 system in this species (S9 Fig). Stable and continuous expression of Cas9 nuclease and gRNA from the 18S rDNA region facilitates complete editing of multiple loci followed by a single (antibiotic resistance gene) integration event in *Leishmania* genome.

## Supporting information

S1 Fig Maximum-likelihood phylogenetic tree of Leishmaniinae reconstructed using *LmM*.*22*.*0010* homolog sequences. Numbers at nodes indicate bootstrap percentage and posterior probability, respectively. Values less than 0.5 and 50% are replaced with dashes. Nodes having 1.0 posterior probability, 100% bootstrap support are marked with black circles. The tree is rooted with the sequence of *Blechomonas ayalai*. The scale bar denotes the number of substitutions per site.
(PDF)

S2 Fig Crispr-Cas9 mediated ablation of mCherry. A, Schematic representation of the *mCherry* locus before and after Puro integration. Arrows indicate relative positions of the primers used for PCR verification. B, PCR confirmation of correct integration. Puro integration into genomic DNA of KO line is confirmed by PCR with primers mCherry_F_NcoI and mCherry_R_NotI (mCherry size is 744 bp; after donor insertion size is 2,354 bp). Cells expressing mCherry (mCh), and CRISR-Cas9 system with U6 promotor in the forward (KO U6+, populations 1 and 2), and reverse (KO U6-, population 1) orientation were analyzed along with the negative control (-C). L, is 1 kb ladder. B, C, Light (left panels) and fluorescent (right panels) microscopy of the representative *L*. *mexicana-mCherry* (B) and *L*. *mexicana-mCherry* KO U6+ p.1 (C) cells. Scale bars are 20 μm.
(PDF)

S3 Fig Crispr-Cas9 mediated ablation of *LmxM*.*22*.*0010*. A, Schematic representation of the *LmxM*.*22*.*0010* locus before and after Puro integration. Arrows indicate relative positions of the primers used for PCR verification. B, PCR analysis of clonal cultures and a negative control. 1 kb DNA ladder is on the left. C, RT-qPCR analysis of clone 1 was done as described in [10]. Data were normalized to *LmxM*.*07*.*0510* [28].
(PDF)

S4 Fig Growth curves for the wild type, Cas9, and BTN1 KO *L*. *mexicana*. Results of three independent biological replicates are presented.
(PDF)

S5 Fig Quantification by RT-qPCR of *Pfr1D*, *Sherp*, and *Amastin* gene expression. These transcripts were used as markers for promastigotes (both pro- and metacyclics), metacyclics, and amastigotes, respectively. Data are from four independent biological replicates (parasites passaged through insects and mice). The error bars indicate standard deviations. Averaged expression values for *LmxM*.*07*.*0510* and *LmxM*.*36*.*1140* ware used for normalization [28].
(PDF)

S6 Fig Development of clinical symptoms in the inoculated mice ears. Diameter of the lesions from three independent experiments measured weekly. The mice ear lesions photos represent situation at the end of experiments (13–16 weeks p.i.).
(PDF)

S7 Fig Number of parasites in the inoculated mice ears infected by WT, Cas9, and BTN1 KO strain of *L*. *mexicana*. Numbers of parasites were determined by qPCR analysis in the end of the experiments (13–16 weeks p.i.). Boxplots are from three independent biological replicates and show 1st quartile, median, 3rd quartile, and 1.5× interquartile range values.
(PDF)

S8 Fig Correlation of lesion size and number of parasites. Quantitative PCR of the *L*. *mexicana* load in the inoculated mice ears in the end of the experiments (13–16 weeks p.i.).
(PDF)

S9 Fig Schematic representation of the modified CRISPR-Cas9 system. Note that a single integration of Puromycin is sufficient for ablation of all copies of the gene of interest because of the continuous gRNA expression. This way, cells with a single integration of the Puro cassette are converted to double-, triple- and quadruple KOs at later stages.
(PDF)

S1 Table List of primers used in this study for amplification of the *LmxM*.*22*.*0010* gene specific targeting sequences, selectable markers, final fusion PCR products used for *L*. *mexicana* transfection, Southern blot probes, and PCR analysis of correct integration. (XLSX)

S2 Table Pairwise sequence comparison of *LmxM*.*22*.*0010* homologs in Leishmaniinae and *Blechomonas ayalai*. (XLSX)

S3 Table Average gene coverage for genes on chromosome 22 of *L*. *mexicana* M379. (XLSX)

S4 Table Morphological analysis of *Leishmania mexicana* cells in sand fly guts 7–8 days p.i. Number of detected morphotypes listed according to *Leishmania* strains and three independent experiments. LN, long nectomonade; SN, short nectomonade; ME, metacyclic promastigote.
(XLSX)
