# Supplementary figures and images for: CRISPR/Cas9 in Leishmania mexicana: A case study of LmxBTN1
Source: PLoS One. 2018 Feb 13;13(2):e0192723. doi: 10.1371/journal.pone.0192723 (PMC5811015; doi:10.1371/journal.pone.0192723)

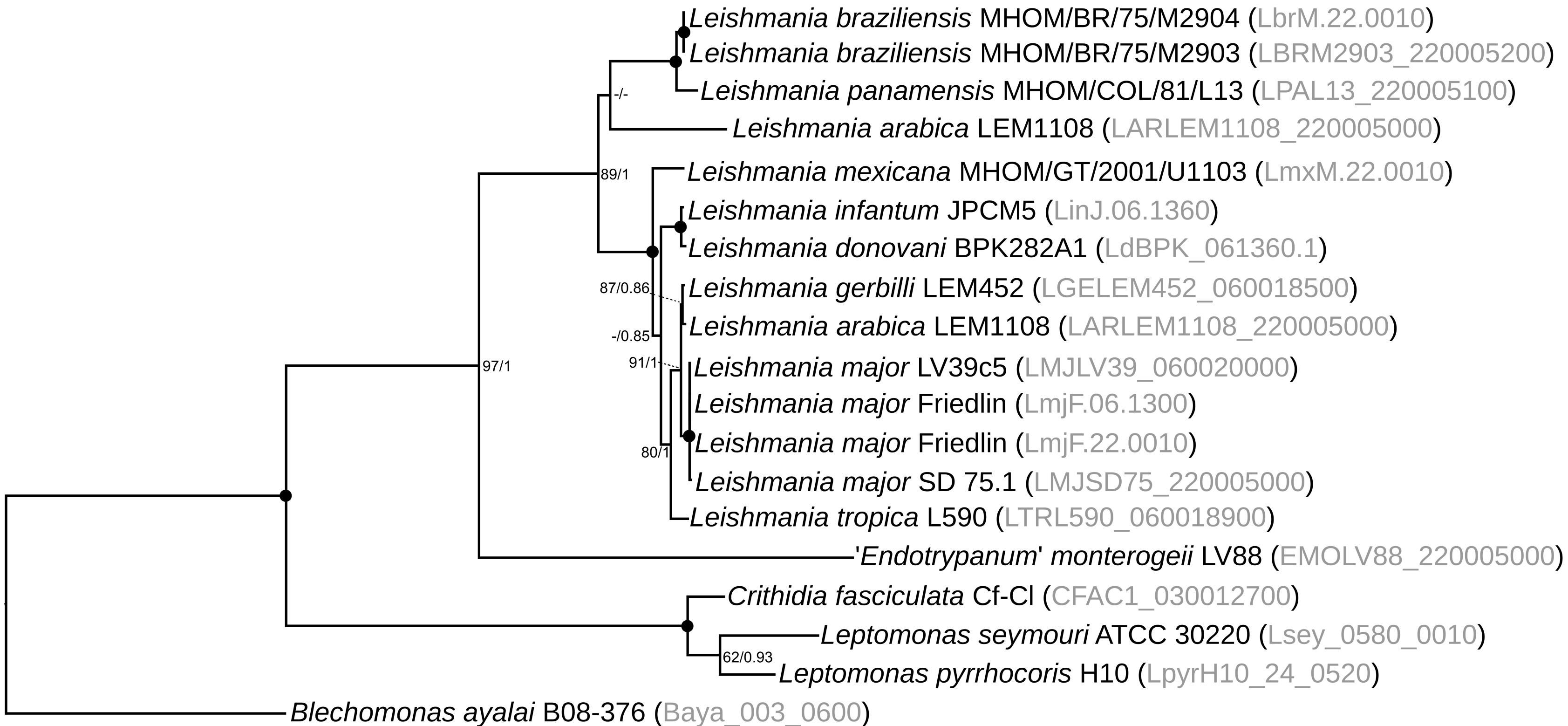

0.2

Supplement: S1 Fig — Numbers at nodes indicate bootstrap percentage and posterior probability, respectively. Values less than 0.5 and 50% are replaced with dashes. Nodes having 1.0 posterior probability, 100% bootstrap support are marked with black circles. The tree is rooted with the sequence of Blechomonas ayalai. The scale bar denotes the number of substitutions per site. (PDF) [file pone.0192723.s001.pdf]

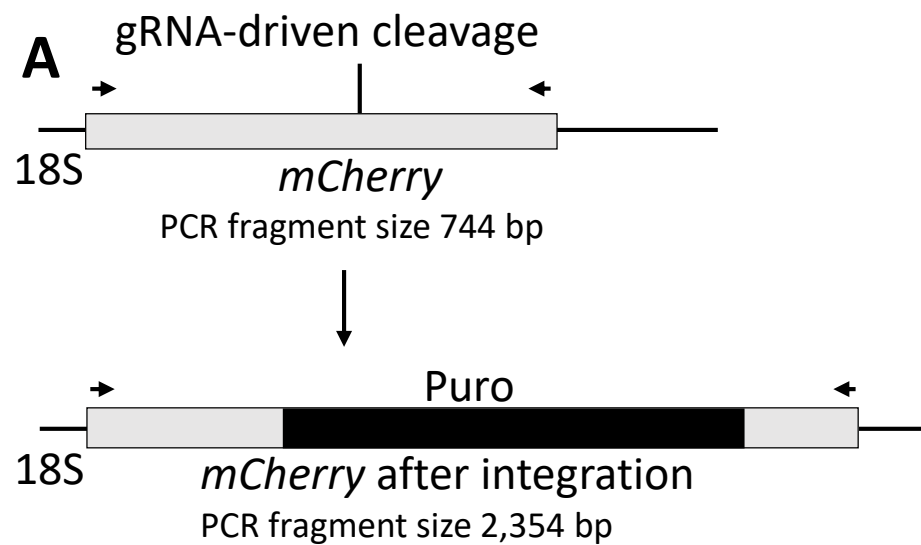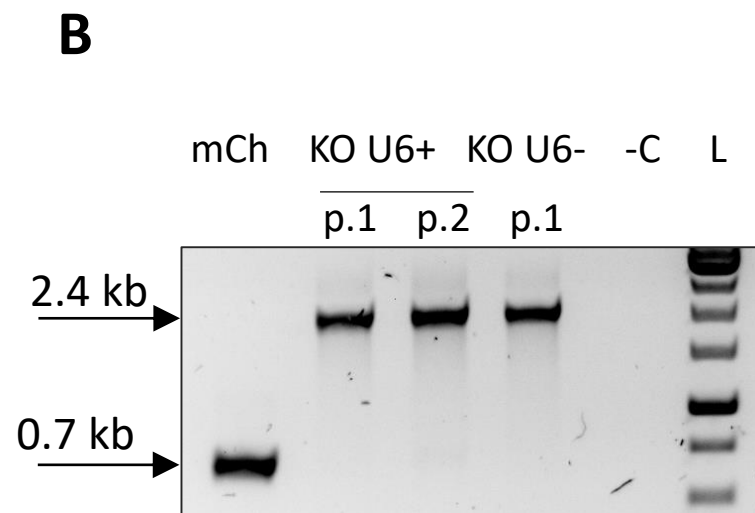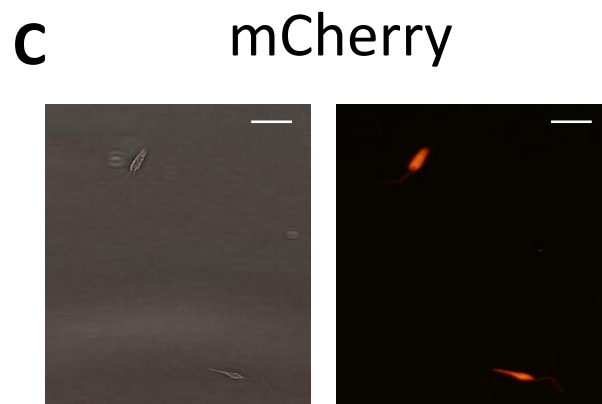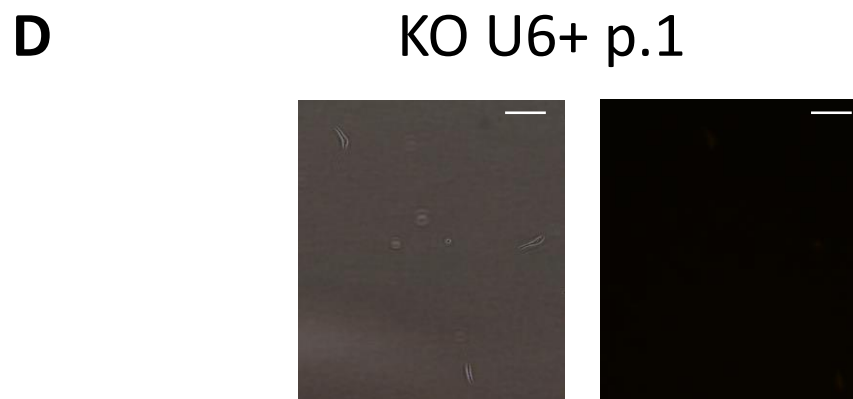

Supplement: S2 Fig — A, Schematic representation of the mCherry locus before and after Puro integration. Arrows indicate relative positions of the primers used for PCR verification. B, PCR confirmation of correct integration. Puro integration into genomic DNA of KO line is confirmed by PCR with primers mCherry_F_NcoI and mCherry_R_NotI (mCherry size is 744 bp; after donor insertion size is 2,354 bp). Cells expressing mCherry (mCh), and CRISR-Cas9 system with U6 promotor in the forward (KO U6+, populations 1 and 2), and reverse (KO U6-, population 1) orientation were analyzed along with the negative control (-C). L, is 1 kb ladder. B, C, Light (left panels) and fluorescent (right panels) microscopy of the representative L. mexicana-mCherry (B) and L. mexicana-mCherry KO U6+ p.1 (C) cells. Scale bars are 20 μm. (PDF) [file pone.0192723.s002.pdf]

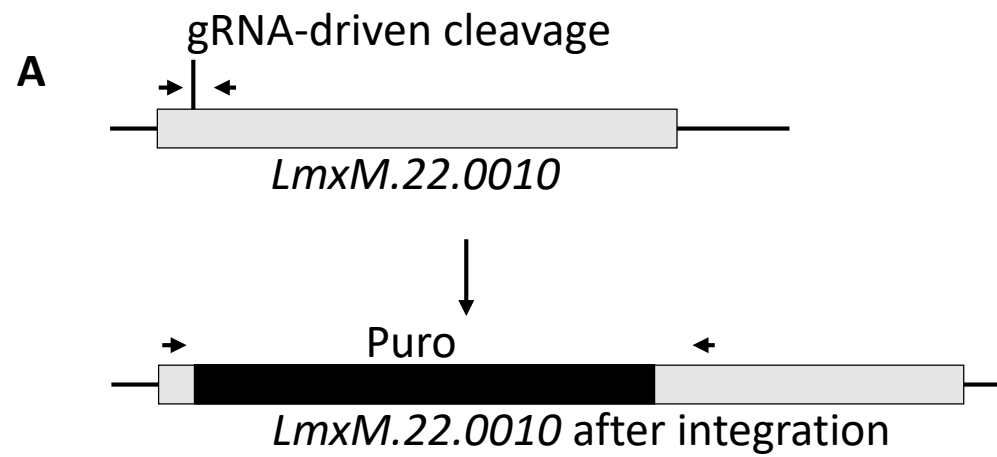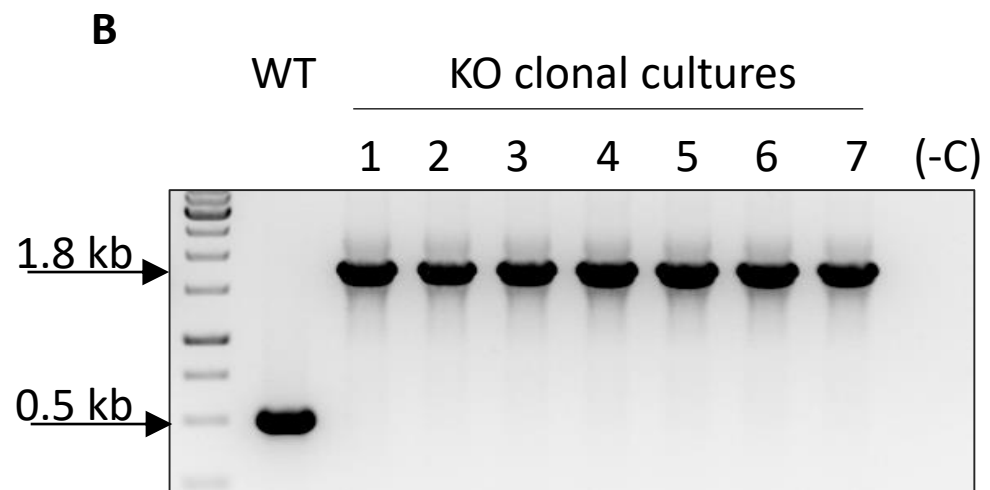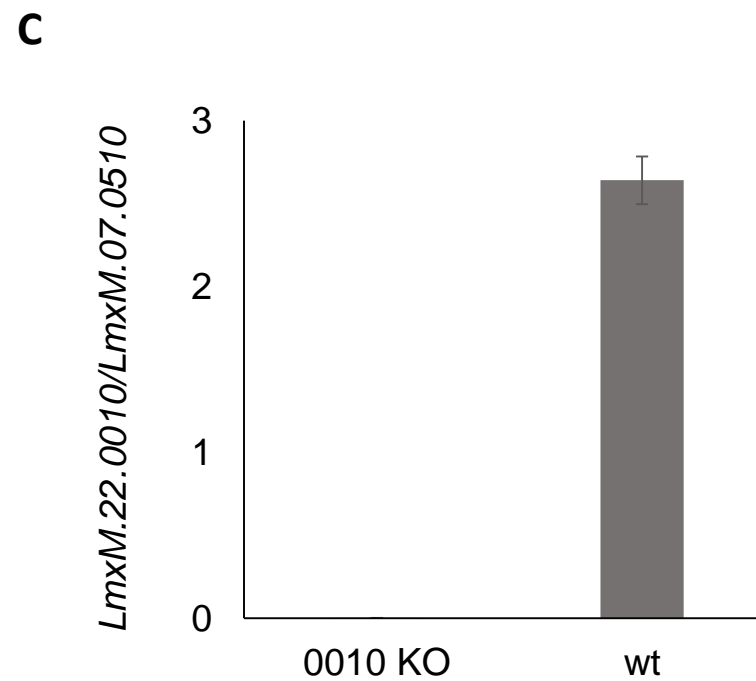

Supplement: S3 Fig — A, Schematic representation of the LmxM.22.0010 locus before and after Puro integration. Arrows indicate relative positions of the primers used for PCR verification. B, PCR analysis of clonal cultures and a negative control. 1 kb DNA ladder is on the left. C, RT-qPCR analysis of clone 1 was done as described in [10]. Data were normalized to LmxM.07.0510 [28]. (PDF) [file pone.0192723.s003.pdf]

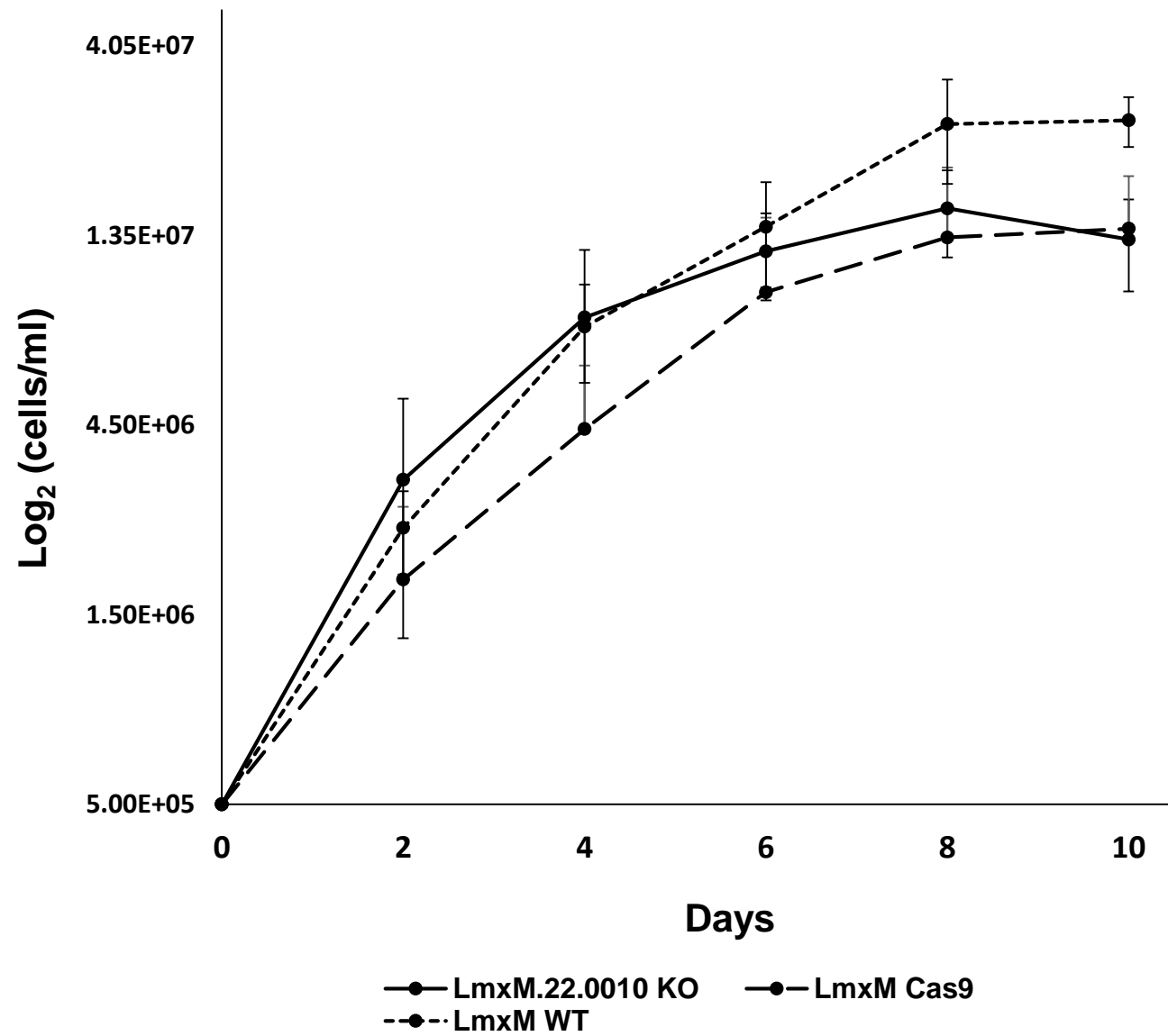

Supplement: S4 Fig — Results of three independent biological replicates are presented. (PDF) [file pone.0192723.s004.pdf]

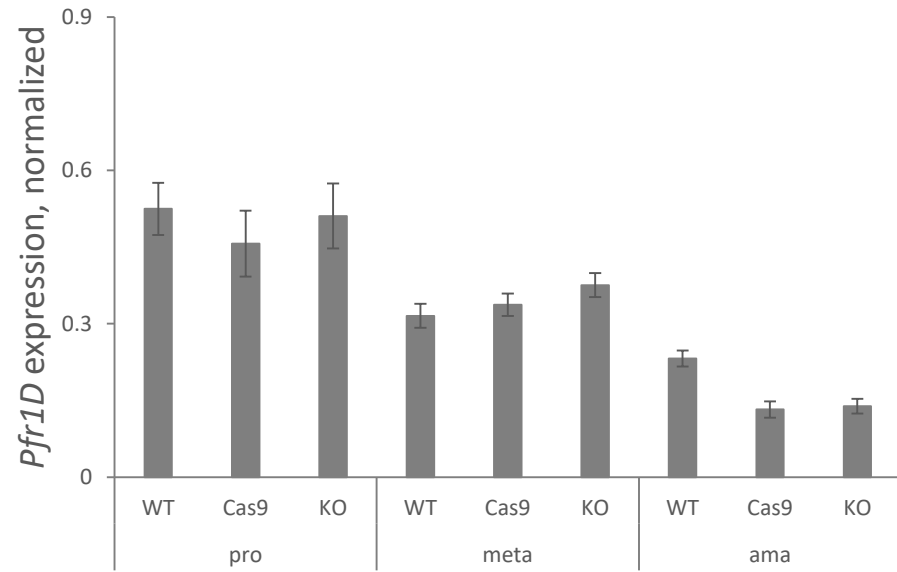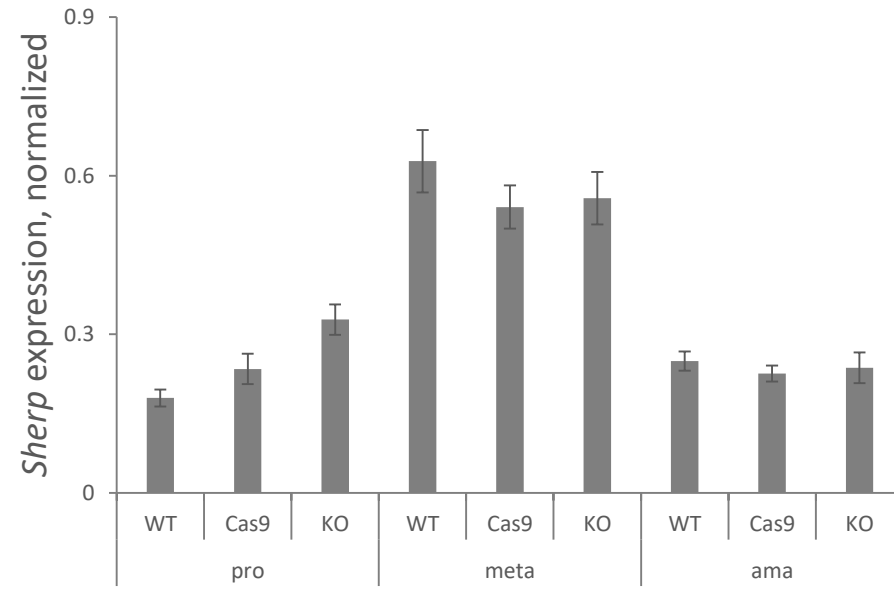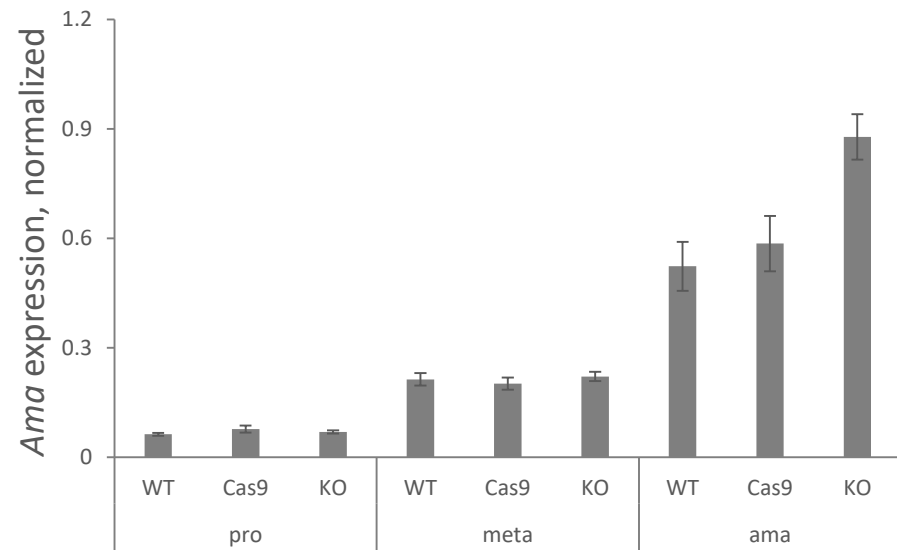

Supplement: S5 Fig — These transcripts were used as markers for promastigotes (both pro- and metacyclics), metacyclics, and amastigotes, respectively. Data are from four independent biological replicates (parasites passaged through insects and mice). The error bars indicate standard deviations. Averaged expression values for LmxM.07.0510 and LmxM.36.1140 ware used for normalization [28]. (PDF) [file pone.0192723.s005.pdf]

WT exp. no. 1

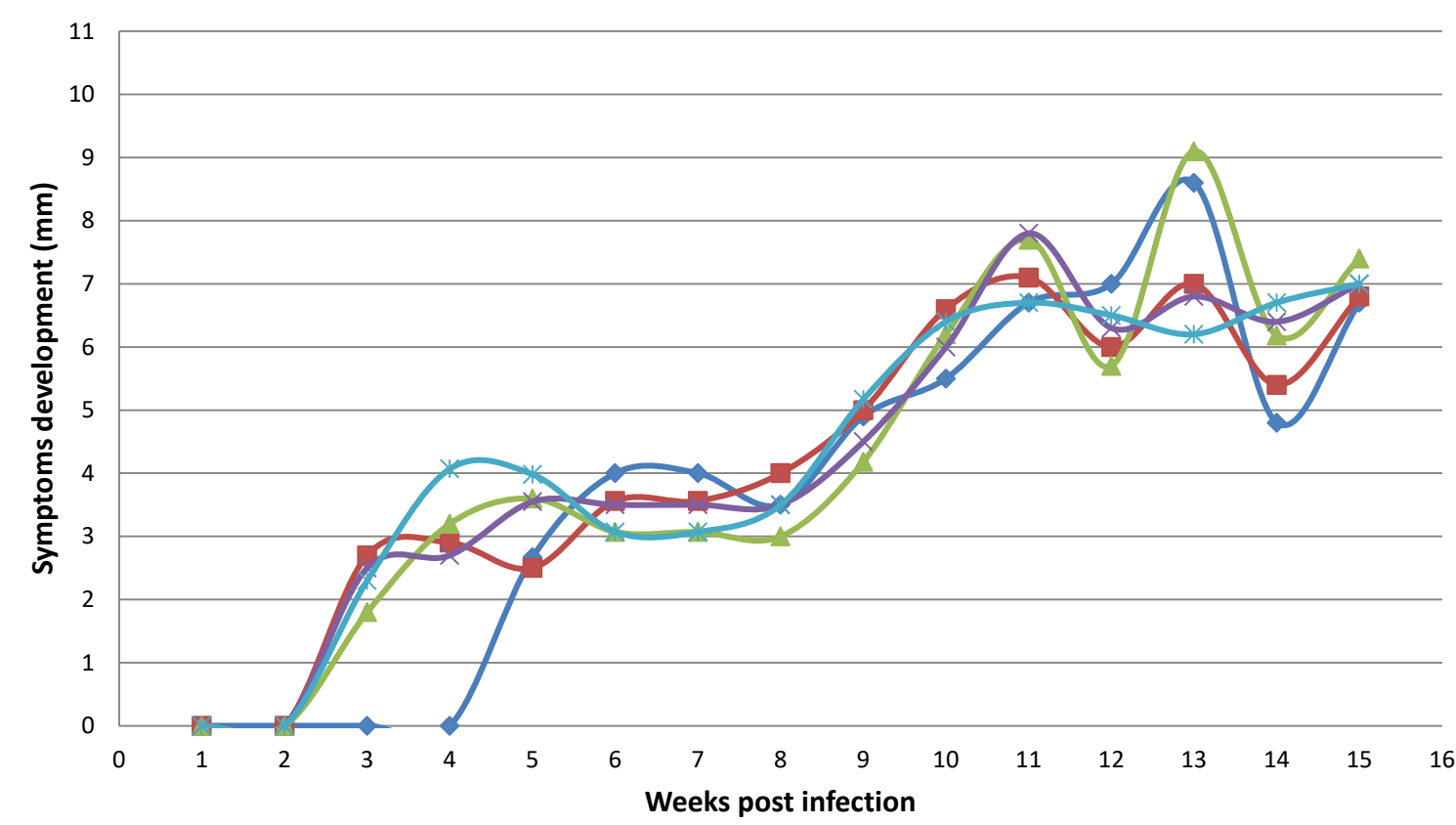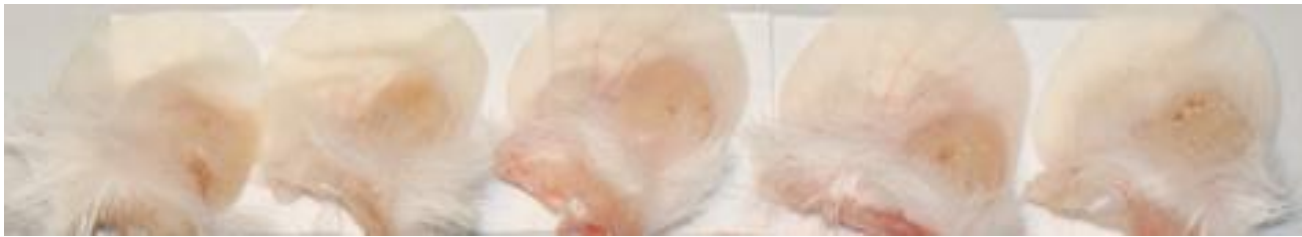

WT exp. no. 2

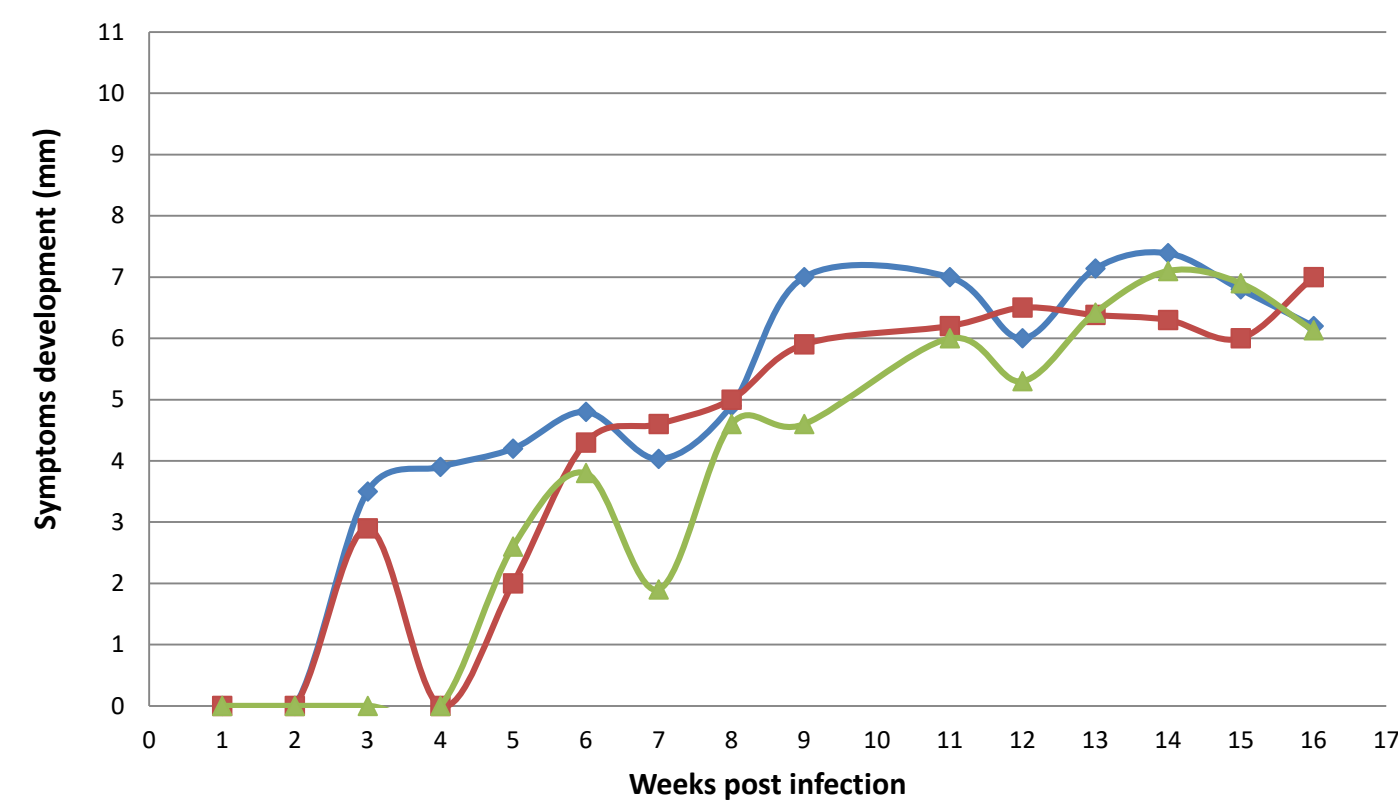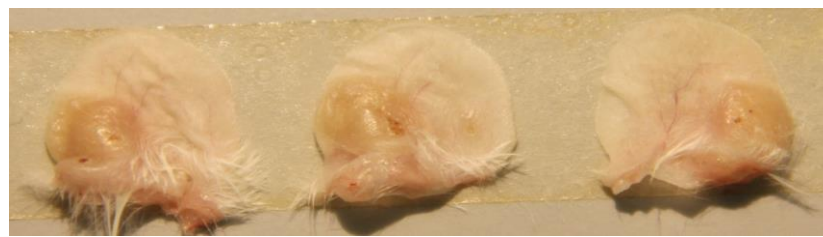

WT exp. no. 3

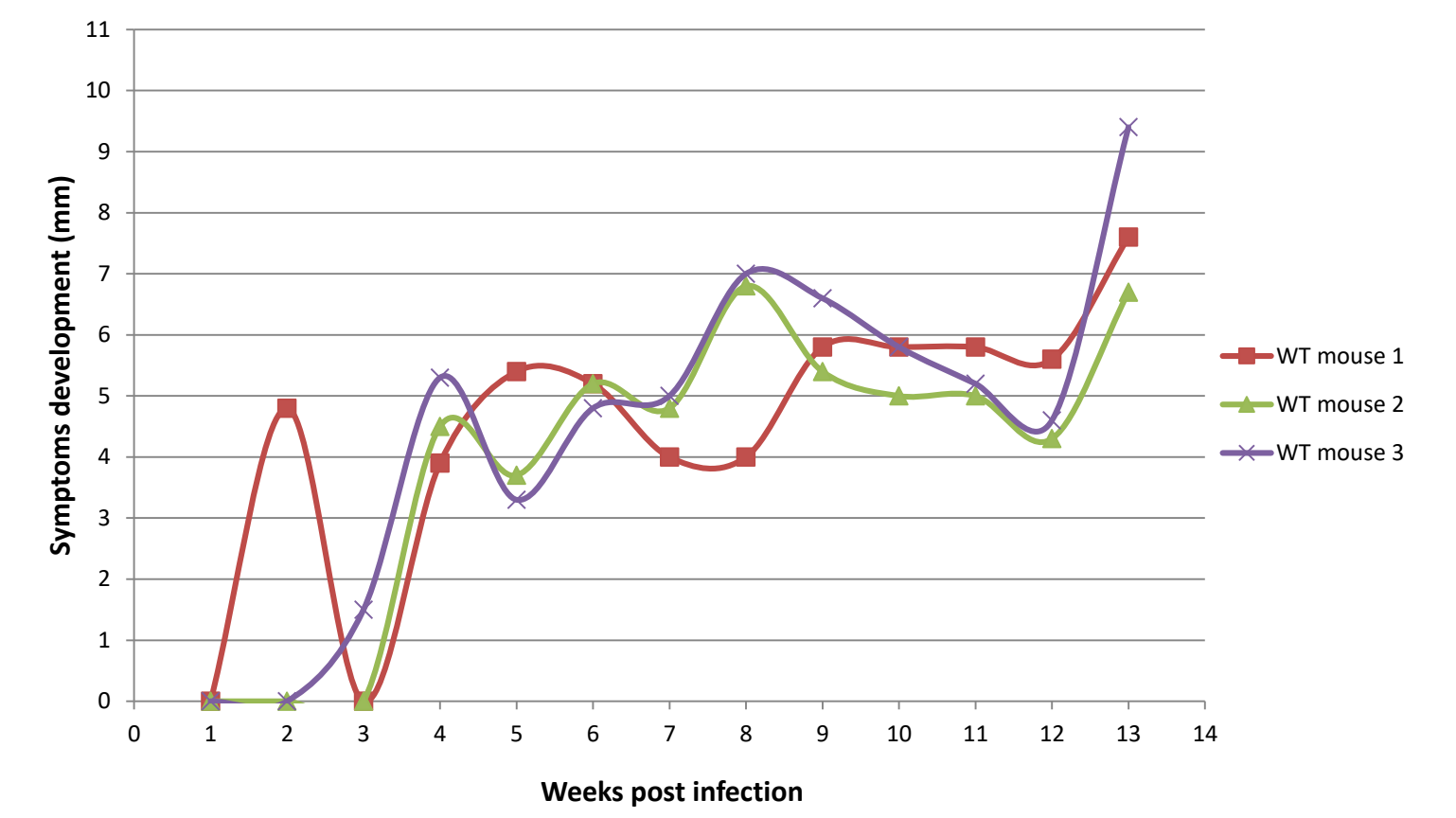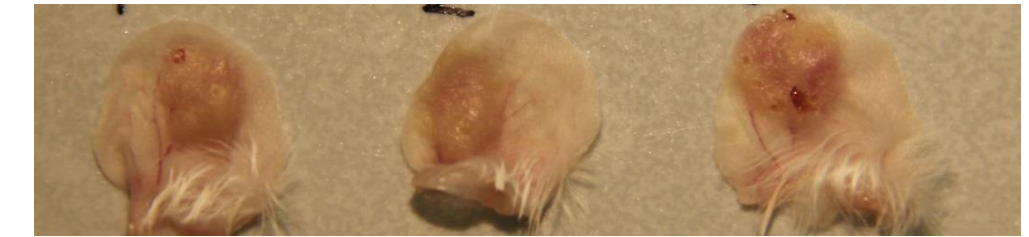

Cas9 exp. no. 1

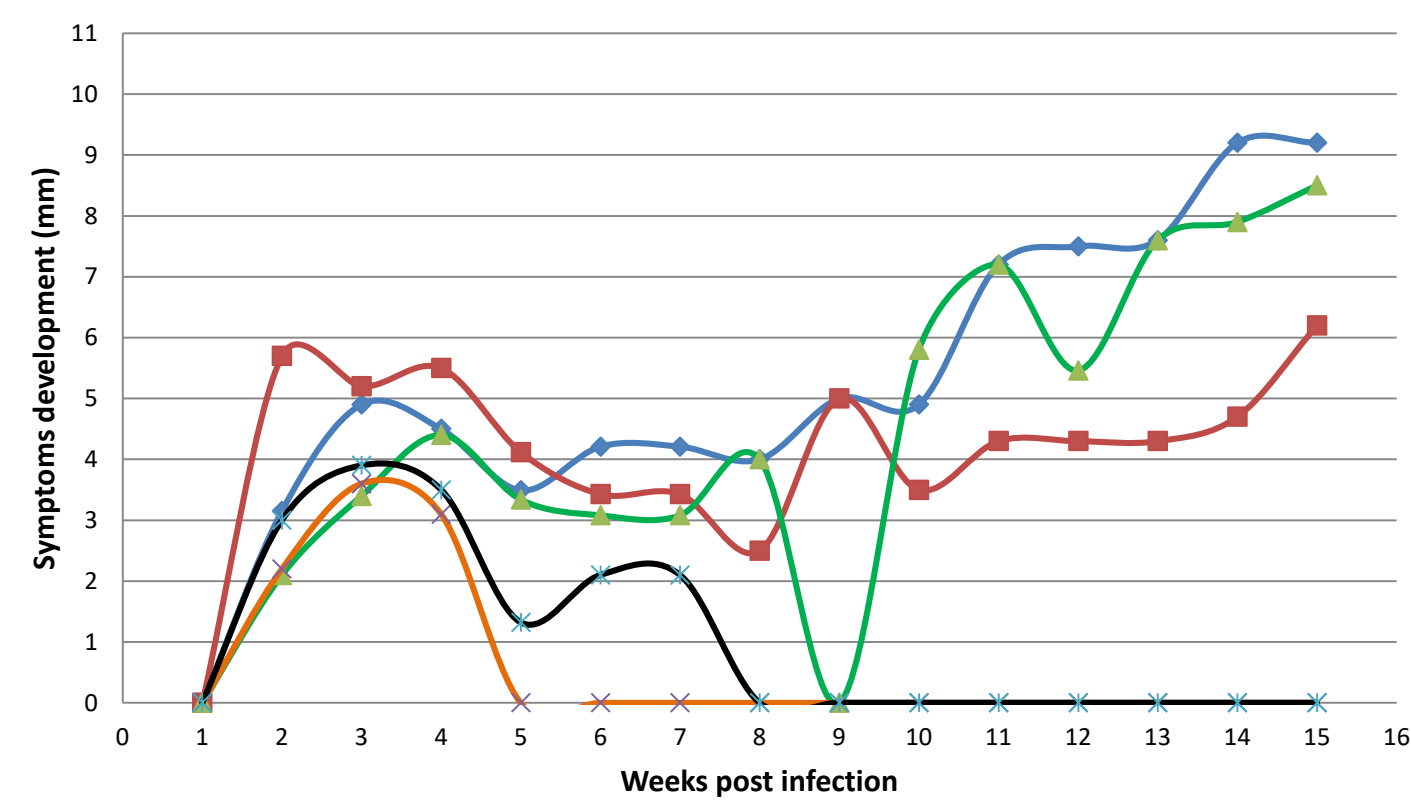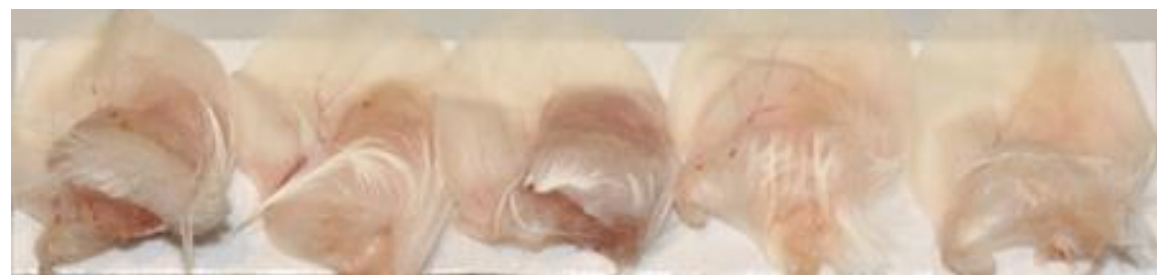

Cas9 exp. no. 2

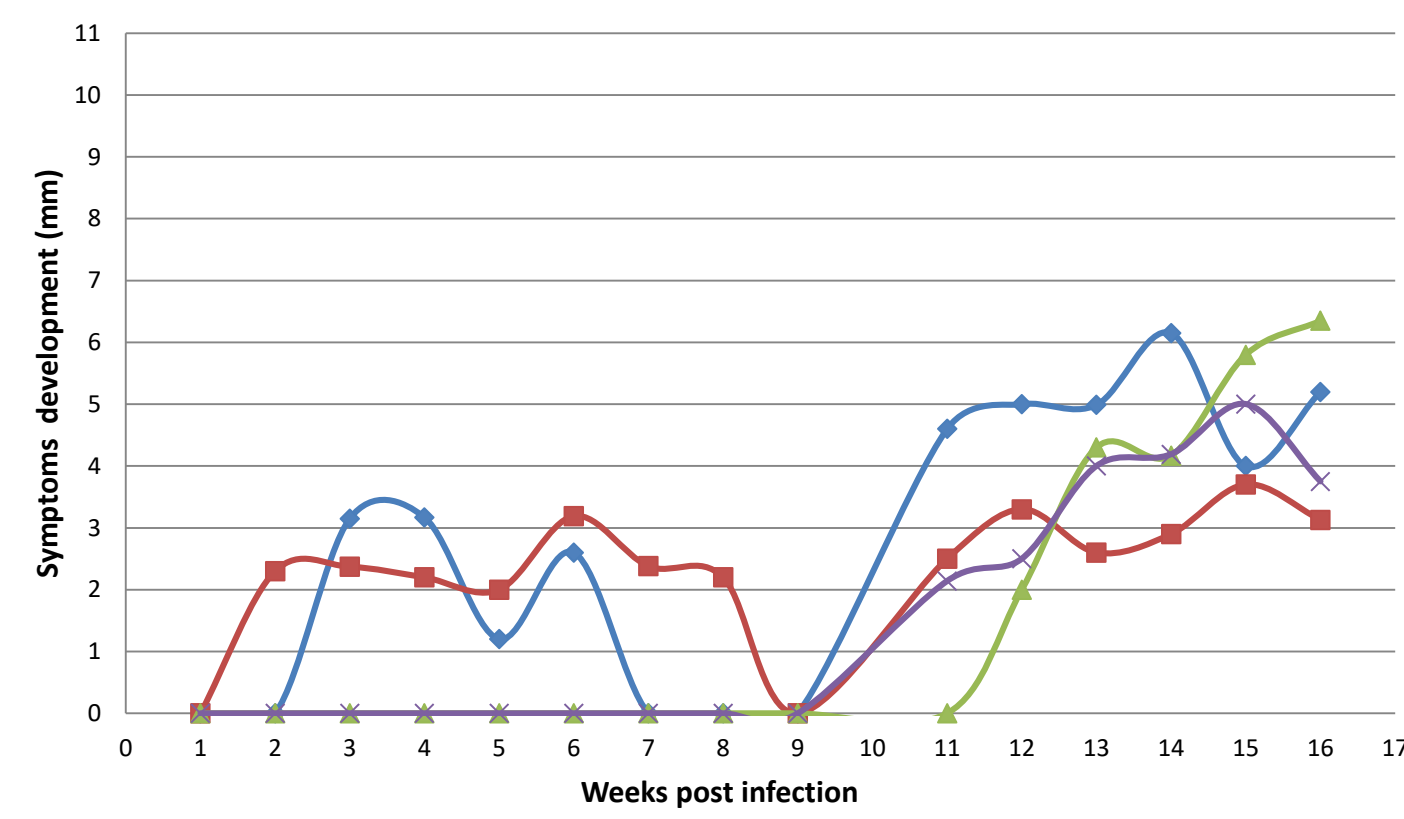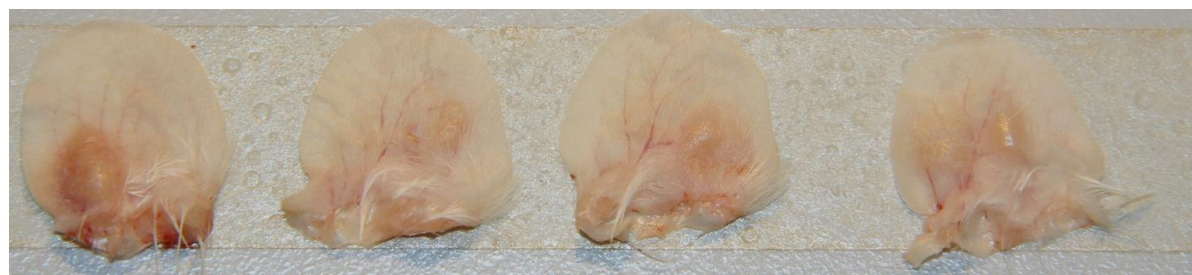

Cas9 exp. no. 3

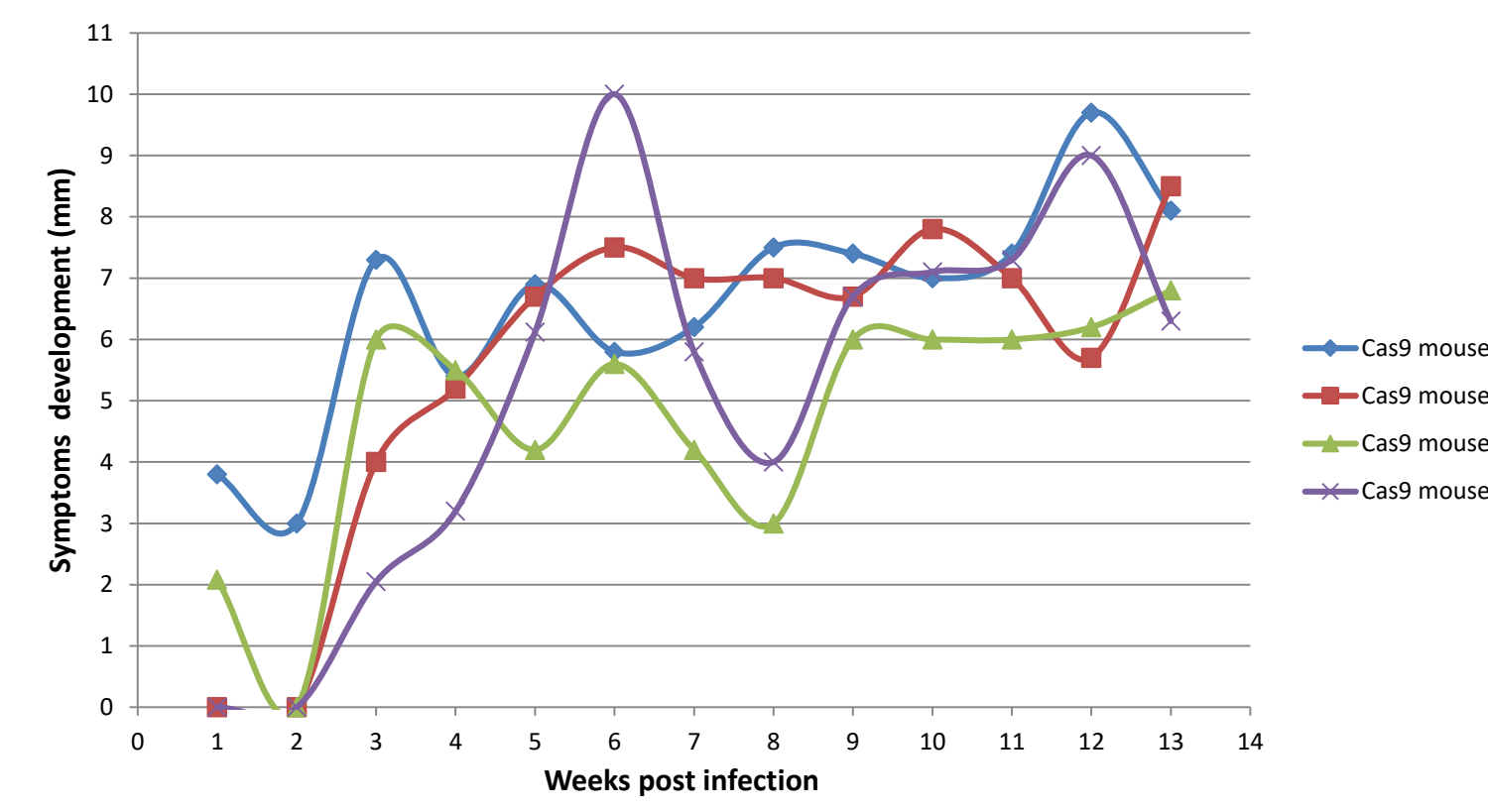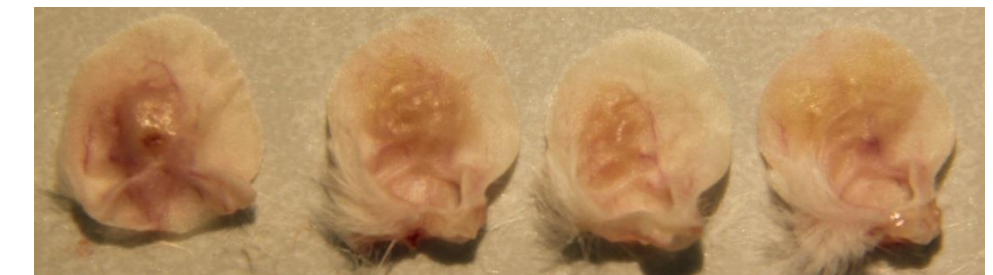

KO exp. no. 1

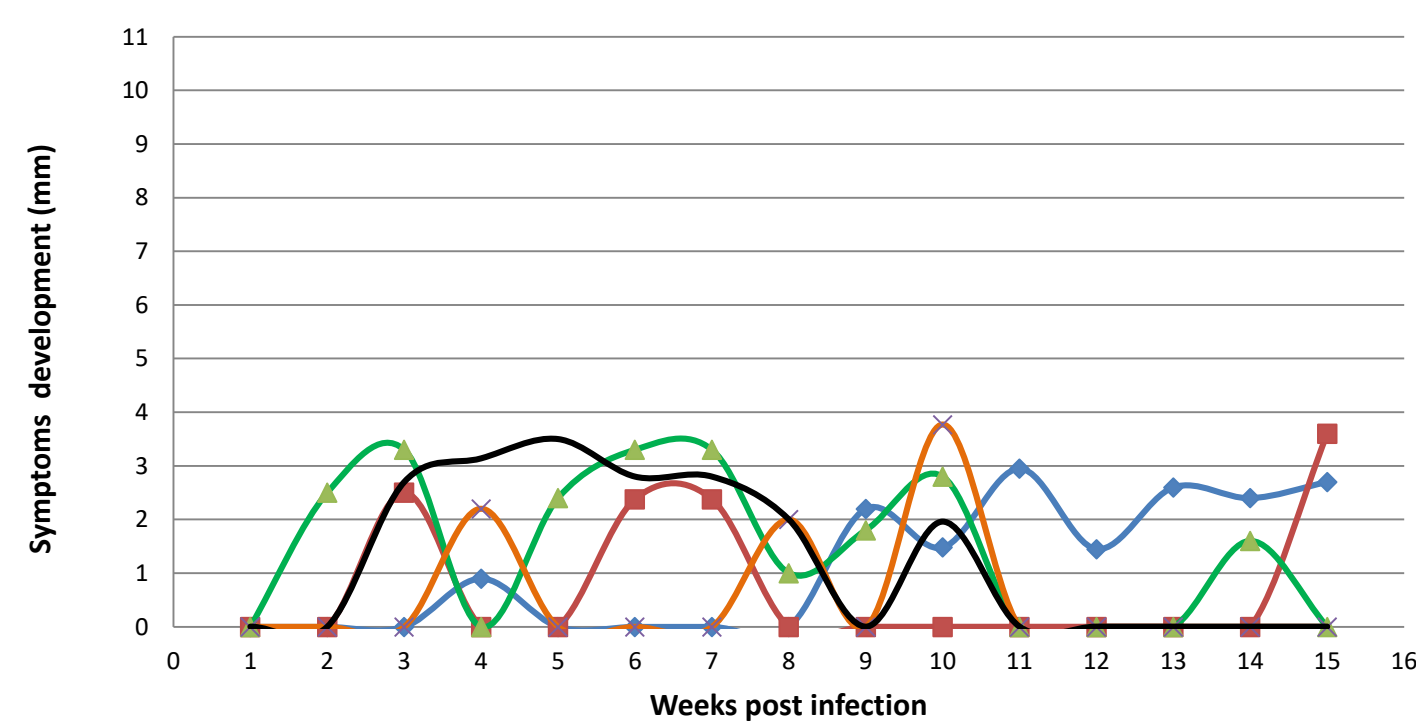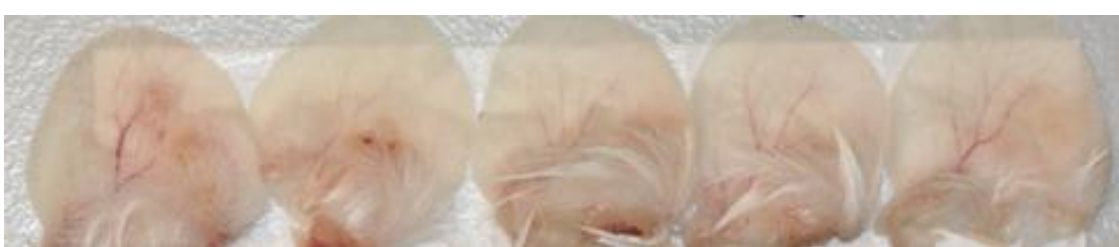

KO exp. no. 2

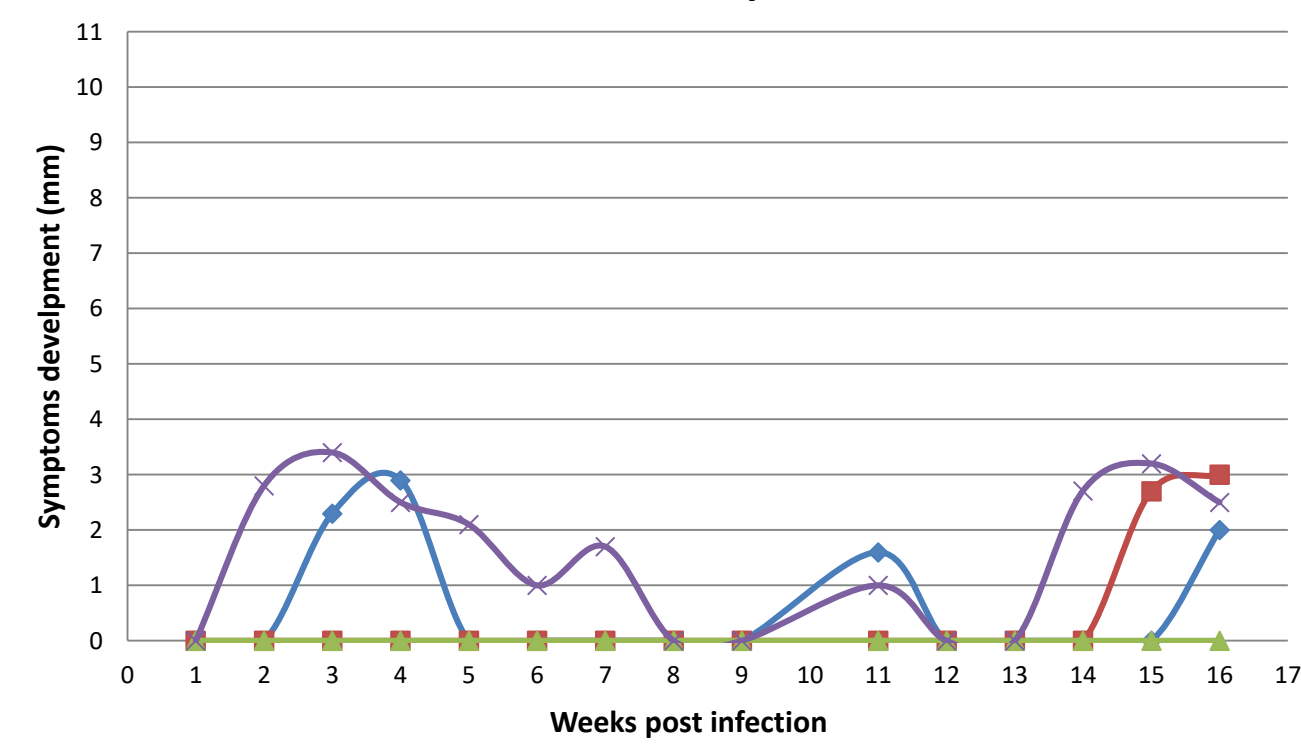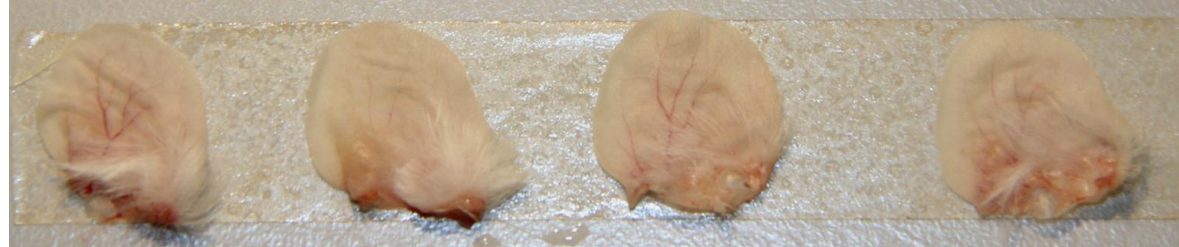

KO exp. no. 3

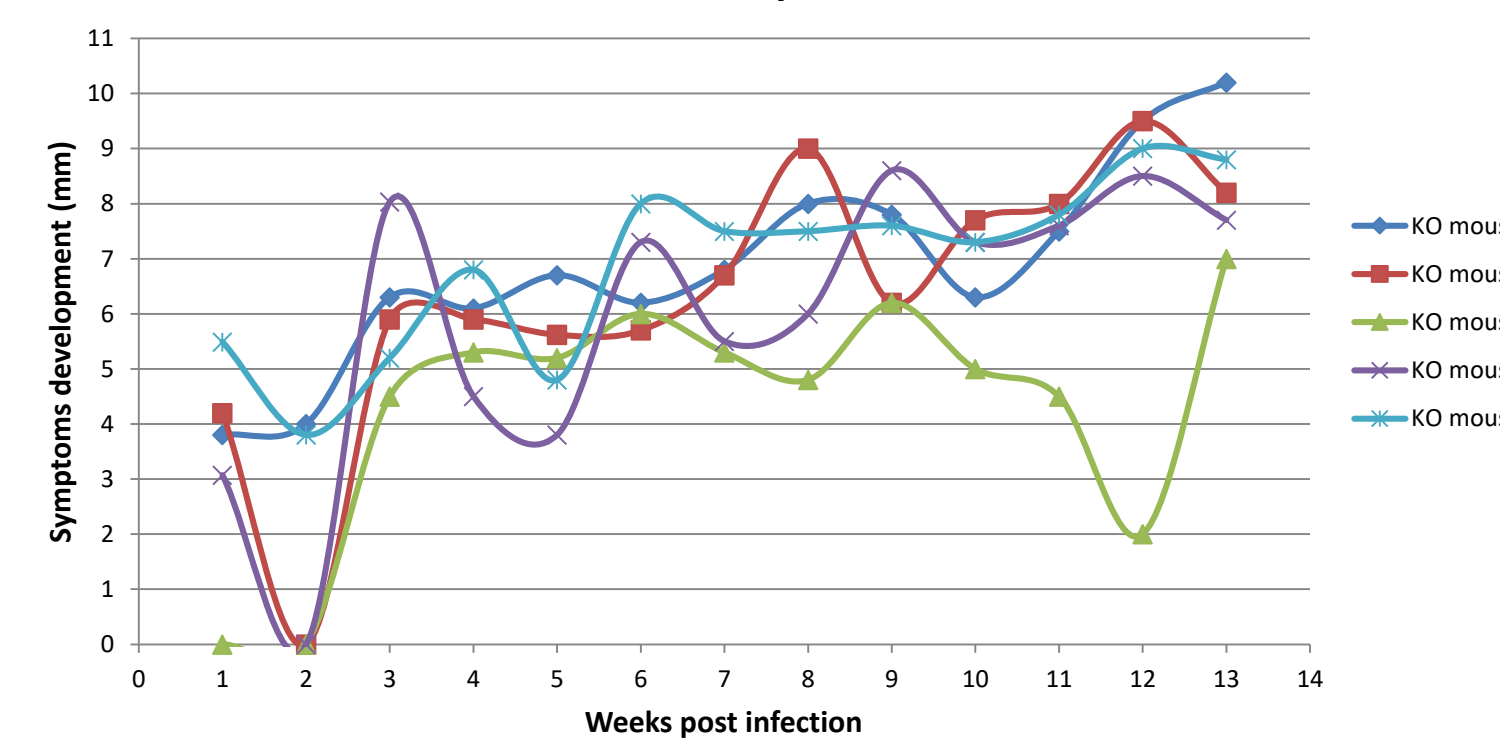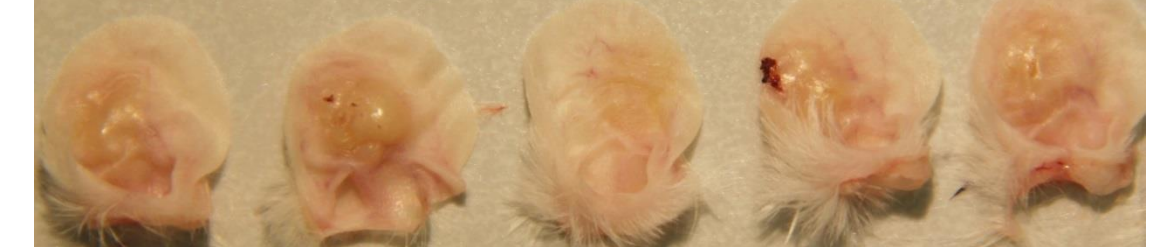

Supplement: S6 Fig — Diameter of the lesions from three independent experiments measured weekly. The mice ear lesions photos represent situation at the end of experiments (13–16 weeks p.i.). (PDF) [file pone.0192723.s006.pdf]

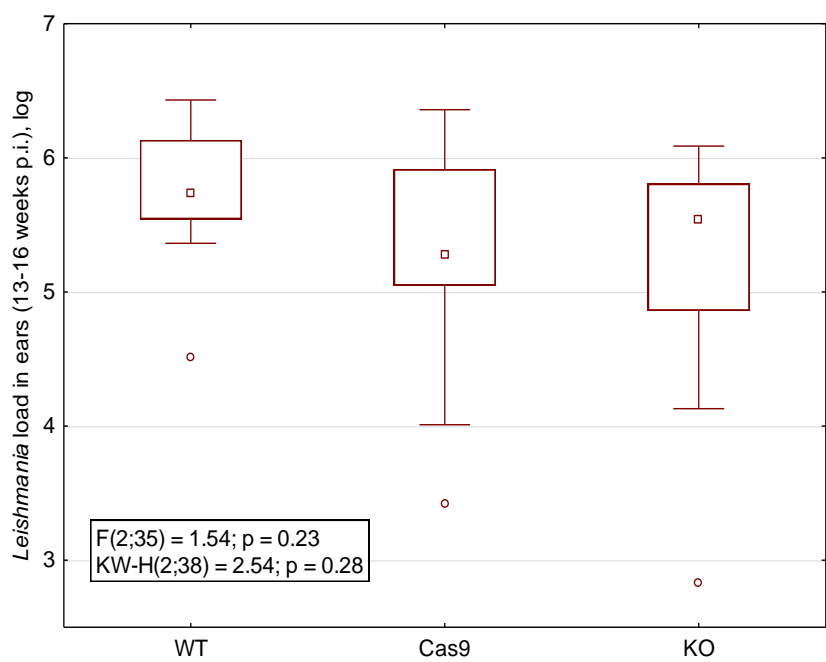

Supplement: S7 Fig — Numbers of parasites were determined by qPCR analysis in the end of the experiments (13–16 weeks p.i.). Boxplots are from three independent biological replicates and show 1st quartile, median, 3rd quartile, and 1.5× interquartile range values. (PDF) [file pone.0192723.s007.pdf]

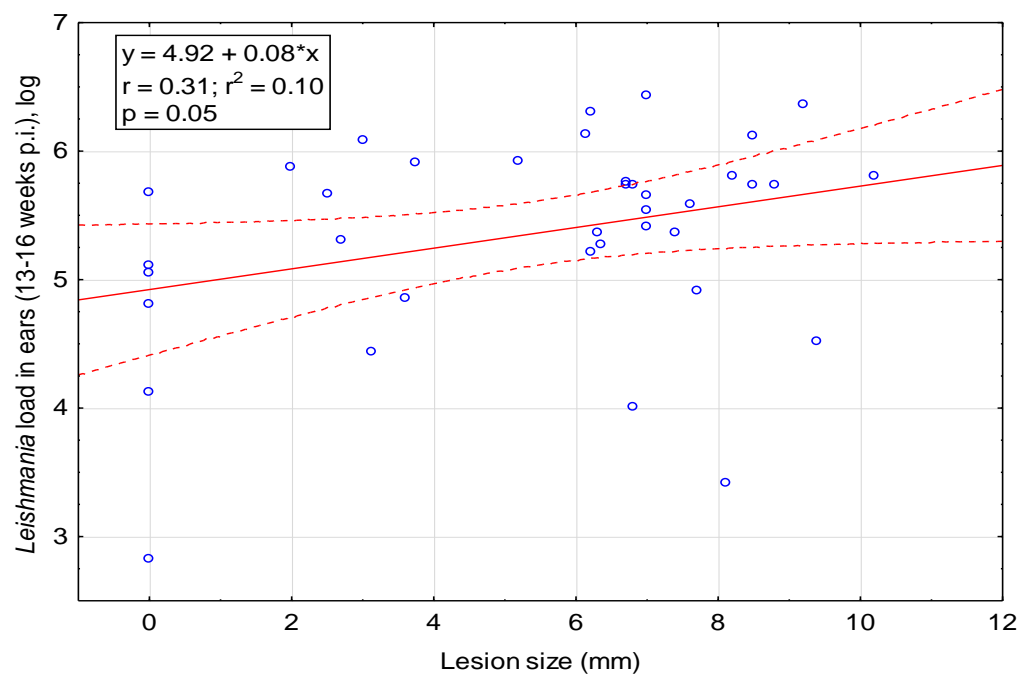

Supplement: S8 Fig — Quantitative PCR of the L. mexicana load in the inoculated mice ears in the end of the experiments (13–16 weeks p.i.). (PDF) [file pone.0192723.s008.pdf]

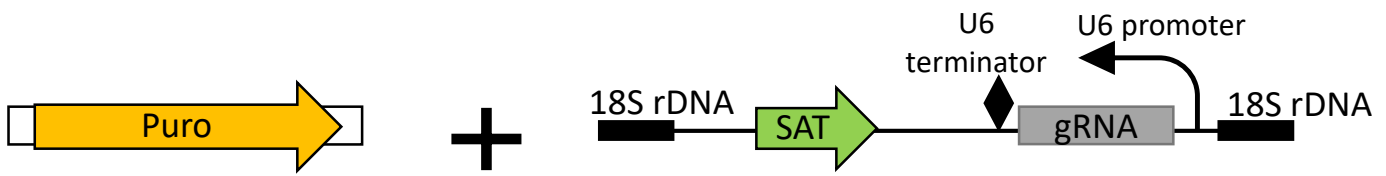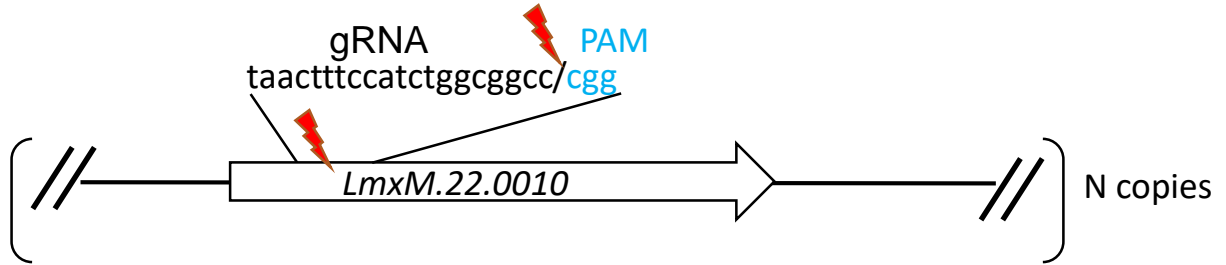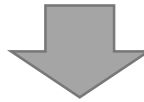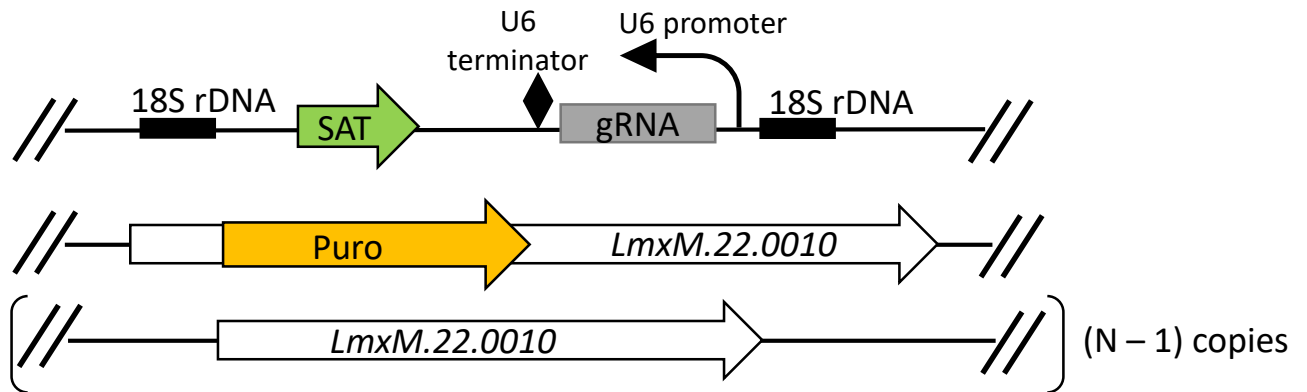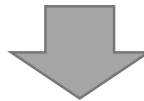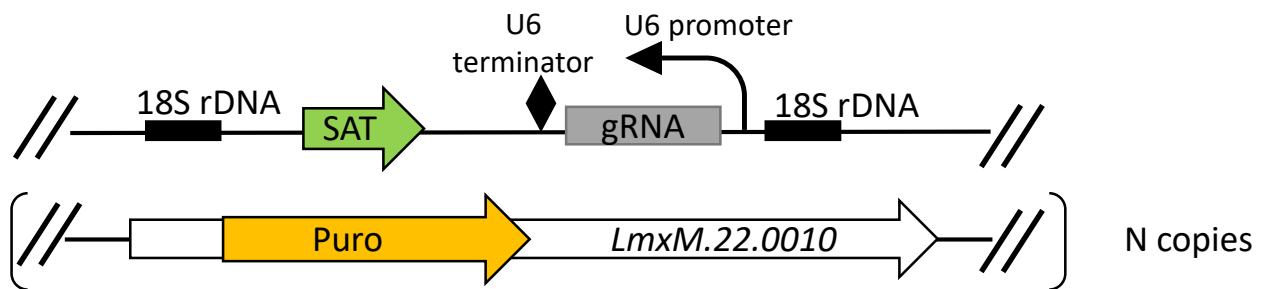

Supplement: S9 Fig — Note that a single integration of Puromycin is sufficient for ablation of all copies of the gene of interest because of the continuous gRNA expression. This way, cells with a single integration of the Puro cassette are converted to double-, triple- and quadruple KOs at later stages. (PDF) [file pone.0192723.s009.pdf]
